# Metabolic Fluxes Using Deep Learning Based on Enzyme Variations: *Application to Glycolysis in Entamoeba histolytica*

**DOI:** 10.3390/ijms252413390

**Published:** 2024-12-13

**Authors:** Freddy Oulia, Philippe Charton, Ophélie Lo-Thong-Viramoutou, Carlos G. Acevedo-Rocha, Wei Liu, Du Huynh, Cédric Damour, Jingbo Wang, Frederic Cadet

**Affiliations:** 1 BIGR, UMR_S1134 Inserm, University of Paris City, 75006 Paris, France; jean.oulia@univ-reunion.fr (F.O.); philippe.charton@univ-reunion.fr (P.C.); ophelie.lo@hotmail.fr (O.L.-T.-V.); 2 Laboratory of Excellence GR-Ex, 75006 Paris, France; 3 DSIMB, UMR_S1134 BIGR, Inserm, Faculty of Sciences and Technology, University of Reunion, 97744 Saint-Denis, France; 4 The Novo Nordisk Foundation Center for Biosustainability, Technical University of Denmark, DK-2800 Kgs. Lyngby, Denmark; cargac@biosustain.dtu.dk; 5 Department of Computer Science and Software Engineering, School of Physics, Mathematics and Computing, The University of Western Australia, Perth 6009, Australia; wei.liu@uwa.edu.au (W.L.); du.huynh@uwa.edu.au (D.H.); 6 EnergyLab, EA 4079, Faculty of Sciences and Technology, University of Reunion, 97490 Saint-Denis, France; cedric.damour@univ-reunion.fr; 7 Department of Physics, School of Physics, Mathematics and Computing, The University of Western Australia, Perth 6009, Australia; jingbo.wang@uwa.edu.au; 8 Artificial Intelligence Department, PEACCEL, 75013 Paris, France

**Keywords:** artificial intelligence, deep learning, metabolic pathway, pathway modeling, deep neural network, flux prediction, glycolysis

## Abstract

Metabolic pathway modeling, essential for understanding organism metabolism, is pivotal in predicting genetic mutation effects, drug design, and biofuel development. Enhancing these modeling techniques is crucial for achieving greater prediction accuracy and reliability. However, the limited experimental data or the complexity of the pathway makes it challenging for researchers to predict phenotypes. Deep learning (DL) is known to perform better than other Machine Learning (ML) approaches if the right conditions are met (i.e., a large database and good choice of parameters). Here, we use a knowledge-based model to massively generate synthetic data and extend a small initial dataset of experimental values. The main objective is to assess if DL can perform at least as well as other ML approaches in flux prediction, using 68,950 instances. Two processing methods are used to generate DL models: cross-validation and repeated holdout evaluation. DL models predict the metabolic fluxes with high precision and slightly outperform the best-known ML approach (the Cubist model) with a lower RMSE (≤0.01) in both cases. They also outperform the PLS model (RMSE ≥ 30). This study is the first to use DL to predict the overall flux of a metabolic pathway only from variations of enzyme concentrations.

## 1. Introduction

Metabolic pathways are interconnected sets of biochemical reactions that enable organisms to produce energy and synthesize molecules essential for their survival. They include processes such as glycolysis, cellular respiration, or amino acid synthesis and are regulated by intricate mechanisms that guarantee their balance and adaptation to the environment. Many bioinformatics applications, such as drug design [1,2,3], synthetic biology [4,5,6], and comparative genomics [7,8,9], depend on understanding these complex metabolic pathways.

For many years, various methods have been developed to model metabolic pathways, including the well-known kinetic models [10]. These models are intended to be more detailed than those based solely on stoichiometry. However, they do have several limitations [11,12]: unknown kinetic parameters of enzymes, differences between in vitro measurements and in vivo conditions, intercellular variability, complex regulation processes, or interactions between different metabolic pathways. Recent advances in in-silico techniques are paving the way for new modeling methods, notably based on Machine Learning (ML) techniques [13]. For instance, ML models outperformed the kinetic model prediction of limonene and isopentenol metabolic pathways [14]. Deep learning is a subfield of ML where multiple layers model process data to extract intricate patterns [15]. There are many different architectures possible that can respond to different types of problems, but the most fundamental model is the Artificial Neural Network (ANN). It is composed of an input layer, one hidden layer, and an output layer. Deep neural network (DNN), which is the type of model used in this study, is an extension of ANN where multiple hidden layers are used. The non-linearity of metabolic pathways can be a major problem, but neural networks are particularly well suited to non-linear phenomena. They have emerged as powerful tools in various research fields [16,17,18,19], including bioinformatics, and are just beginning to be applied to metabolic pathways, enabling accurate predictions of flux [20,21,22,23] and insights into cellular behavior [24,25]. Notably, studies such as those by S. Choudhury et al. [26,27] and X. Wang et al. [28] have demonstrated the potential of DL and particularly neural networks in addressing challenges across metabolic and enzymatic research, including the prediction of dynamic properties of an entire metabolic network, parameterization of kinetic models, and annotation of enzyme active sites. The work of Choudhury et al. [26,27], in particular, focuses on *Escherichia coli*, a well-characterized organism with extensive available data, which contrasts with our study that applies DL methods to a less-characterized system, aiming to explore new predictive capabilities.

**Figure 1:**
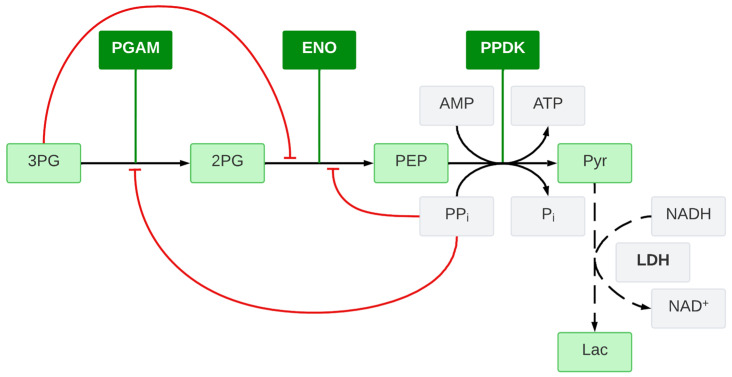
The glycolytic metabolic pathway of *Entamoeba histolytica* drives the conversion of glucose into energy and essential biomolecules. 3-phosphoglycerate mutase (PGAM), enolase (ENO), and pyruvate phosphate dikinase (PPDK) are the three enzymes involved in the metabolic pathway. Red arrows indicate retroinhibition of PGAM and ENO enzymes by 3PG and PPi.

The glycolytic pathway shown in Figure 1 is a pivotal metabolic route driving the conversion of glucose into energy and essential biomolecules in the parasite *Entamoeba histolytica* [29]. Acquiring a comprehensive understanding of this pathway provides valuable insights into the parasite’s mechanisms for survival and potential targets for therapeutic intervention. The second part of glycolysis plays a crucial role by synthesizing essential molecules towards energy production. In the lower part of glycolysis, pyruvate, a pivotal metabolite, is generated through a sequence of enzymatic reactions. It involves the conversion of 3-phosphoglycerate (3-PG) to 2-phosphoglycerate (2-PG) by the enzyme 3-phosphoglycerate mutase (PGAM), followed by the dehydration of 2-PG to phosphoenolpyruvate (PEP) catalyzed by enolase (ENO). Subsequently, pyruvate phosphate dikinase (PPDK) facilitates the transfer of two phosphate groups, a first one from PEP to adenosine monophosphate (AMP), forming pyruvate (Pyr), and a second one from inorganic pyrophosphate (PP_i_) to adenosine diphosphate (ADP), forming adenosine triphosphate (ATP). These reactions contribute significantly to energy generation and the provision of metabolites, ultimately supporting the parasite’s vital physiological processes and growth.

In this study, we aim to address the challenges posed by the limited knowledge and difficulties of kinetic modeling (white-box modeling) through the exploration of a novel approach: a data-driven ML method employing a DNN model based on the enzymes involved in the metabolic pathway. More specifically, the aim is to obtain a predictive model capable of determining the output flux solely from the combination of enzyme concentrations. By focusing exclusively on enzyme concentrations, this approach circumvents the need for other data types, such as metabolite concentrations or kinetic parameters, which may not be readily available for less studied or more complex metabolic pathways. This focus ensures broader applicability of the method across diverse biological contexts. Lo-thong et al. addressed this problem using various ML techniques such as quantile regression forest (QRF) and cubist models but not DNN models. These studies also included a basic ANN that performed less well than models not based on the neural networks mentioned above [20,30]. Thus, it would then be interesting to determine whether deep learning models can achieve results as good as, or even better than, the models used previously, both in terms of prediction accuracy and data processing time. Furthermore, since the studied model only reflects a portion of the metabolic pathway, it is crucial to investigate the use of DNN models, which are capable of handling large datasets, in order to integrate the missing data from the glycolytic pathway and thus model and predict the flux of the entire pathway. For several years, approaches based on deep learning have been growing rapidly. This is due to more accessible computational resources, but also more versatile deep DL architecture. DL models automatically extract useful features from datasets and yield better results than other ML techniques when processing huge amounts of complex data [31,32]. For example, DL models have been developed to predict the turnover number of enzymes using protein sequences or structure [33,34]. A recent study developed an interpretable DNN to predict cell growth phenotype and genetic interaction in eukaryotic cells [35]. Since non-linear systems can be difficult to model, DL approaches are particularly suitable for those systems [36]. Patruno et al. [21] showed that DNN models can be used to predict relative fluxes in yeast metabolism, even when there is only a random subspace of input features. They used metabolite concentrations rather than enzyme concentrations and employed a kinetic model where enzymatic reactions are described only by the mass action law instead of detailed kinetic equations.

The performance of DL models is closely related to the quantity and quality of the data and also to the choice of datasets used in the training and evaluation phases. To assess the performance of the model, it is useful to have a set of results obtained using different training and evaluation procedures. To this end, we have set up two methods in which the generation of the training and validation sets are not identical: cross-validation and the repeated holdout method.

We present a novel approach to modeling metabolic pathways, utilizing advanced DL methods. Our contributions include (i) the development of robust models representing metabolic pathways and (ii) the design of two distinct workflows to evaluate the models’ generalization capabilities across varied conditions. A thorough comparison of the DL models is conducted to assess their respective efficacies. The discussion will include the insights gained, potential shortcomings, and potential industrial applications of the proposed methods.

To the best of our knowledge, this is the first use of DL to predict the overall flux of a metabolic pathway from variations of enzyme concentrations.

## 2. Results

### 2.1. Evaluation of a Simple Neural Network

Table 1 provides a summary of the hyperparameters chosen for the DNN model from a grid search hyperparameter tuning. Find in SI an in-depth explanation for each hyperparameter, including Supplementary Figures S1 and S2 and Supplementary Tables S1 and S2. The model is made up of three hidden layers of 105 neurons each with ELU as the activation function and a single neuron in the output layer, making a total of 22,786 parameters to train. This satisfies our rule of thumb of having fewer trainable parameters than instances in the dataset. The MSE is the chosen loss function for this model, and the parameters optimization is made by the Adam algorithm. The RMSE tells us how accurately a regression model can predict the value of the response variable in absolute terms, while R^2^ tells us how accurately a model can predict the value of the response variable in percentage terms; percentages are more useful for comparing different models on different data sets, but the regression model is optimized using the RMSE. For the training process, the upper limit of epochs is 3000 with a batch size of 100. An EarlyStopping function is set up to monitor the RMSE on the validation set with a patience of 100 epochs.

The training dataset is split into two different sets: the training set (80%) and the validation set (20%). Model parameters are only optimized using the training set. In each epoch, the model evaluates the validation set, and the results are used to detect whether the model is in an overfitting state or not. Being in an overfitting state means that the model has learned “too much” on the training set and has a poor generalization capability. A sign of overfitting is when a model has impressive results on the training set but performs poorly on never-seen data such as the validation set.

Once the training is over, the model is evaluated on the test set initially set aside to obtain its performance on never-seen data. A similarity can be found between the validation set and test set, as they are both technically never-seen data for the model. However, often we select a model based on its performance on a validation set, and this implies indirect use of the dataset for the model. While the validation set is only used to pick the best possible model, it is with the performance on the test set that we can truly analyze the generalization capabilities of the model.

**Figure 2:**
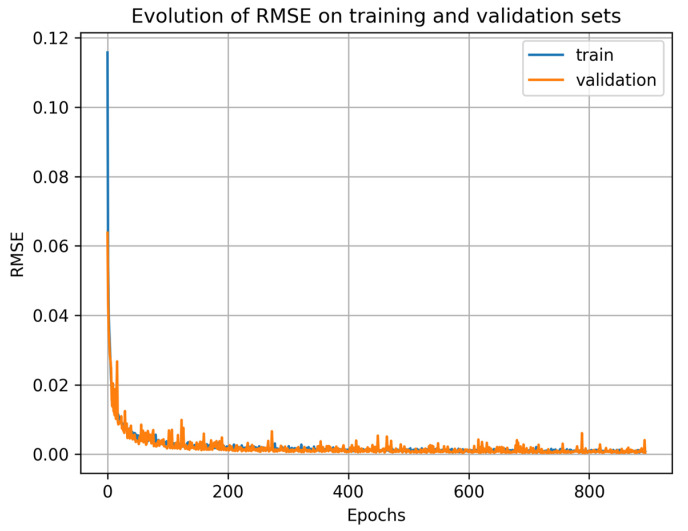
Evolution of the RMSE on the training and validation data over the training epochs. The RMSE values shown in this figure are calculated using the normalized output. The number of epochs was set to 3000, and the RMSE values stop at epoch 894 since the RMSE has not decreased for 100 epochs. After this interruption, the model parameters at epoch 794 were reloaded.

Figure 2 shows the evolution of RMSE, both on the training dataset and the validation dataset, during each epoch of the training process. Results stopped at epoch 894 while the number of epochs was fixed at 3000, showing that the EarlyStopping function has stopped the training process because the RMSE did not improve on the validation set for 100 epochs.

Table 2 is a summary of the results obtained by the model at epoch 794 on the training and validation sets during the training and on the test set during the evaluation. During the training process, metrics were computed using normalized values (expected values and predicted values). When computing the metrics in Table 2 below, we have de-normalized both the ground truth and the prediction. The performance on the validation set and test set are close, demonstrating that the model has a good generalization capability and is unlikely to overfit.

With this defined model architecture, we test two different approaches to assess their impact on performance.

### 2.2. Repeated Cross Evaluation Procedure

In this approach, the test set is divided into five subsets to better test the generalization capacity of the models. After isolating the test sets, a cross-validation is performed to estimate the model performances: five models are trained and evaluated using 5-fold cross-validation. The cross-validation procedure is repeated 10 times to generate, train, and evaluate a total of 50 models. Section 4.2.4 gives more details about this approach.

Models’ performance on RMSE, R^2^, and MAE tends to be similar between the validation set and the five test sets. On the validation set, the average RMSE is 0.096, and the best models have an RMSE lower than 0.09 and can reach 0.081. On test sets 1, 4, and 5, the mean RMSE is lower than 0.09, and the best ones reach 0.69. The results are slightly worse on test set 3 with a mean RMSE of 0.96. The performance on test set 2 is comparatively inferior to the other sets, with models yielding a mean RMSE of 0.133 but remaining close. Find more detailed results in Table 3 along with results with R^2^ and MAE. The R^2^ of 0.999 is consistent between the validation and test sets, and the MAE mirrors this. See Supplementary Figures S3–S5 to visualize RMSE, R2, and MAE results. Regarding those results, it is safe to say that the models have great generalization capability with close results on the validation set and the different test sets. See in Supplementary Figures S6 and S7 the difference between the predicted and observed output flux J_pred_ from the validation set and each test set. Displaying little difference, these plots further validate the generalization capabilities of models.

It could be interesting to compare test sets against each other to see if performance is consistent. By taking the results of the test sets two by two, we can use the following statistical tools: Kendall’s tau [37], Spearman’s rank correlation [38], and Pearson’s correlation coefficient.

Each of these metrics produces a value between −1 and 1, which quantifies the correlation between the results in two test sets. By computing these metrics on every combination of test sets, we can obtain an overview of the consistency of each model; e.g., if a model has the best performance on one test set, is it likely to have the best performance on the other test set? We use a heatmap to visualize these correlation metrics.

**Figure 3:**
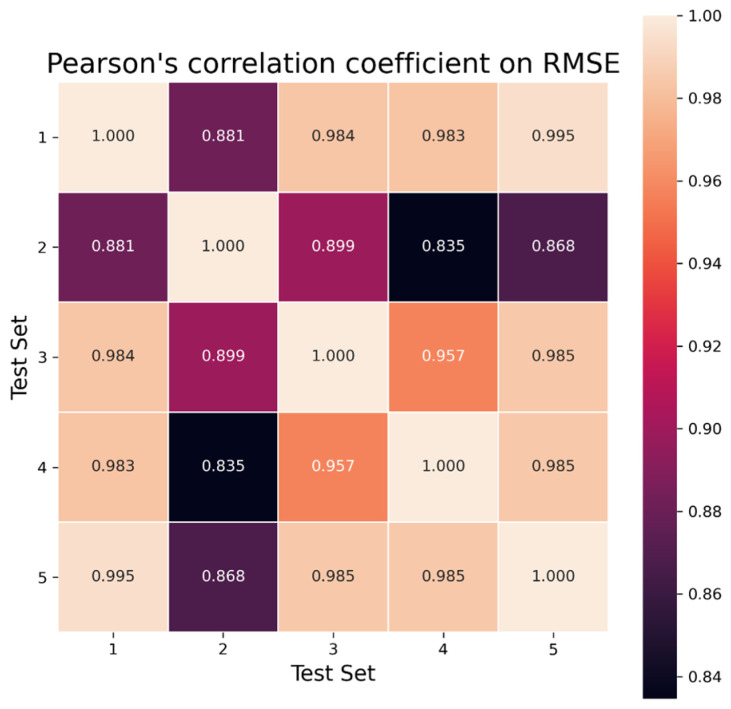
Heat map representing the Pearson’s correlation coefficient on the five test sets (numbered from 0 to 4) during repeated cross-validation for RMSE. The heat map of Pearson’s correlation coefficient for R^2^ is in Supplementary Figure S8 and MAE in Figure S9. The correlation of the RMSE results between the different test sets is very strong (always higher than 0.8): The models have a good generalization capacity.

We can see in Figure 3 the Pearson’s correlation coefficient on RMSE between every combination of test sets, and in the Supplementary Material, the Pearson’s correlation coefficient on R^2^ (see Supplementary Figure S8) and MAE (see Supplementary Figure S9). There is a strong correlation between the results obtained on RMSE, R^2^, and MAE since the calculated correlations are all larger than 0.9. Heat maps for Kendall’s tau (Supplementary Figures S10–S12) and Spearman rank correlation (Supplementary Figures S13–S15) can be found in the Supplementary Material. All Pearson’s correlation coefficients have a value over 0.83, with Kendall’s Tau peaking at 0.983 on RMSE and Spearman’s rank correlation reaching 0.992 on RMSE. The correlation metrics values obtained on the test sets are substantially similar, implying that the models generated have a consistent performance between each set.

### 2.3. Repeated Holdout Evaluation Procedure

Similar to the previous procedure, the test set is divided into five test subsets to test the generalizability of the models, as the size of the data allows. However, the training and validation data in this procedure are randomly generated from the rest of the data. Section 4.2.5 provides more details for this approach.

In order to conduct a relevant comparison with the results obtained with cross-validation, we set up a repeated holdout evaluation with shuffling to generate, train, and evaluate 50 different models. Similarly, models obtained with this approach have close performance between the validation set and the test sets. See in Table 4 results on RMSE, R^2^, and MAE. The mean RMSE on the validation set, with 0.101, is close to the means of test sets 1, 3, 4, and 5 (ranging between 0.089 and 0.098). Here also the performance on test set 2 is the worst one. The coefficient of determination R^2^ is the same in each set. The MAE mean is 0.063 on the validation set and ranges between 0.061 and 0.066 on the other test sets. Analogously with cross-validation results, find in Supplementary Figures S16–S18 plots to visualize metrics performance on the validation set and the test sets. These models, generated with the repeated holdout procedure, also have a good generalization capacity. See Supplementary Figures S19 and S20, where the difference between the predicted and observed target is shown for the validation set and the test sets. For each set, the difference is minimal and empirically approves the generalization capabilities of these models.

**Figure 4:**
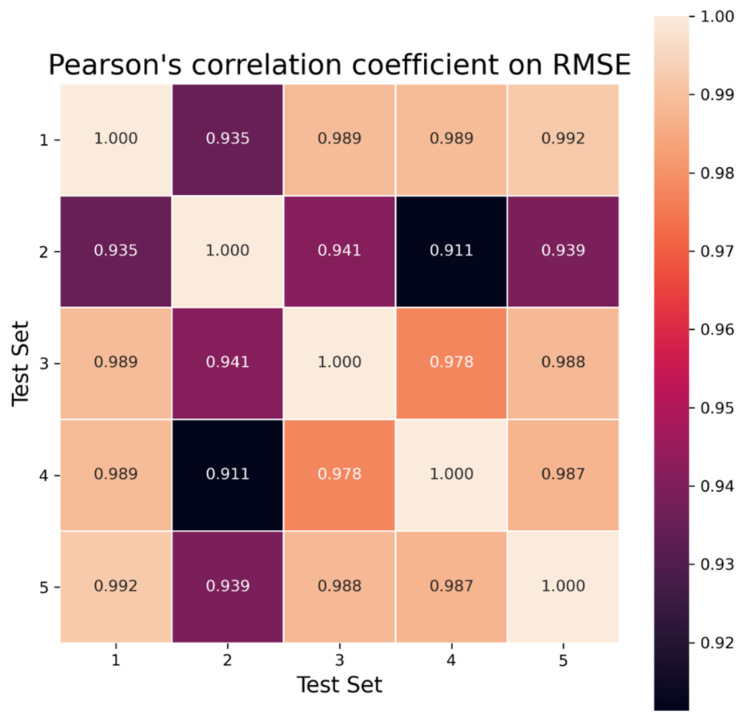
Heat map with the Pearson’s correlation coefficient on the RMSE results obtained on the test sets with the repeated holdout evaluation procedure. Pearson’s correlation coefficient results for R^2^ are available in Supplementary Figure S21, and Pearson’s rank correlation results for MAE are available in Supplementary Figure S22. The correlation of the RMSE results on the different test sets is strong: there is a good generalization capacity of the models that have been trained.

As with the previous procedure, heat maps are used to visualize Pearson’s correlation coefficient on RMSE, MAE, and R^2^ between the different test sets (see Figure 4 for RMSE and see Supplementary Figure S21 for R^2^ and Supplementary Figure S22 for MAE). The heat maps of Kendall’s tau and Spearman’s rank correlation are given in Supplementary Material (Supplementary Figures S23–S28). There is a strong correlation between the results obtained by each metric on the test sets, as the Pearson’s correlation coefficient for the RMSE varies between 0.911 and 0.992.

### 2.4. The Two Procedures Lead to Similar Results

To generate the training set and validation set with the repeated cross-validation method, a constraint is added for each fold to respect the distribution of the initial dataset. On the other hand, the repeated holdout evaluation uses randomness to generate the training set and validation set: with this method, there is no control of the distribution.

**Figure 5:**
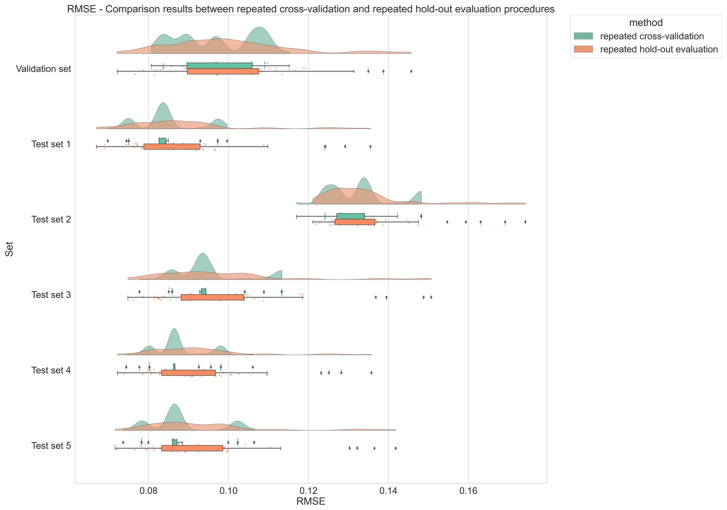
Overlay of the RMSE boxplots obtained during the two procedures (repeated cross-validation and repeated holdout evaluation) in order to better observe the differences in performance, if any. For each set, results are represented through a density wave with a boxplot below it, and the diamonds represent outlier results. Find in green the performance of cross-validation models and in orange repeated holdout evaluation models. The average performance on each set is close between each approach (difference less than 0.01), but the repeated hold-out evaluation approach has more dispersed results. Similar plots for R^2^ are available in Supplementary Figure S29 and for MAE in Supplementary Figure S30.

Results produced by the two procedures are very close to each other: Figure 5 shows a graph where the boxplots obtained with the two procedures are superimposed to better observe the common points and the differences in performance. For each evaluated set (the validation set and the five test sets), RMSE results obtained on the two approaches represented by the density waves (green for repeated cross-validation and orange for repeated holdout) have a great overlap, suggesting that similar models are generated with each approach. However, there are some discrepancies that can be noticed between the two procedures. Results obtained with repeated cross-validation tend to be more concentrated, with 75% of models having RMSE results within a 0.02 space (except on the validation set). On the other hand, results from repeated hold-out evaluation are more dispersed. While some models have a lower RMSE than the other approach, there are more models with worse results. This can be noted by the long-shaped density wave or the diamonds on the boxplots. See Supplementary Figure S29 for R^2^ comparison and Supplementary Figure S30 for MAE comparison.

The similarity between the two approaches can also be noted through performances from Table 3 in Section 2.2 and Table 4 in Section 2.3. RMSE means from repeated hold-out evaluation are always slightly higher but still very close, with a maximum difference lower than 0.005. The worse performances on test set 2 can be seen in the two tables. Better results on RMSE can be found in Table 4, except on Test Set 2, but are not significantly better than results in Table 3 (difference of 0.009 on the validation set and maximum difference on any test set: 0.003). However, worse results are also found in Table 4 on RMSE. The RMSE range upper limits found in Table 4 are 0.036 or 0.038 higher than the results in Table 3. Similar behavior can be spotted with MAE performance, but they are less significant. R^2^ results are identical between the two approaches. More decimals are needed to see the difference, but it shows that the difference is not significant.

The *t*-test is a parametric statistical test tool to be used when we want to know if data sets are similar or not. This tool indicates whether it is possible to reject the null hypothesis that the results have an identical mean by producing a *p*-value, a positive number ranging from 0 to 1. If this *p*-value is higher than the selected significance level, we cannot reject the null hypothesis that the two means are identical. In our case, with a significance level of 0.05, the *p*-values are much higher on test sets 2 and 3 and slightly higher than the threshold on test set 1. On test sets 4 and 5, the *p*-values are lower. On each test set, it is always the results produced by the repeated cross-validation procedure that have the best mean (see in Table 5 *p*-values between each test set). Thus, the difference between the two procedures is not statistically significant for the test set 1 to 3.

## 3. Discussion

The main goal of this study is to accurately predict the output flow, from the variations of enzyme concentrations involved in a metabolic pathway, with a deep neural network. The glycolysis metabolic pathway is used as an application example. We used two different approaches to obtain prediction models on the Glycolysis dataset: cross-validation and repeated holdout evaluation.

Workflows of both methods are quite similar, first setting aside a test set that remains the same throughout the process. However, the ways of generating the training set and validation set differ in each workflow. In cross-validation, the different folds are generated under constraint: they must respect the distribution of the initial set as much as possible in order not to bias the results. In repeated holdout evaluation, the initial dataset is shuffled before being split into a training set and a validation set. If the initial database is not large enough, there is no guarantee that the generated sets respect the distribution of the initial set. In both workflows, the test set, needed to evaluate the generalization capabilities, is created by a random split from the initial dataset, and in our case, the two generated subsets respect the distribution of the original dataset. The two methods used in this paper generated multiple models (50 models each) to better expose the performance variations.

Both approaches provide very similar results since the means of RMSE, R^2^, and MAE in Table 3 and Table 4 are close, as the *t*-test values in Table 5 suggest. Additionally, the slightly inferior performance observed on test set 2 (highest RMSE) may highlight subtle differences in data distribution or features that could merit closer examination to understand the model’s limitations. Nevertheless, by inspecting the range of RMSE results in Table 3 and Table 4, disparities can be seen. While repeated cross-validation gives more concentrated results, the repeated hold-out evaluation approach gives more dispersed results. Better results can be found with a lower boundary, but worse results can also be found with a higher upper limit value. This difference in behavior is more easily spotted through Figure 5, where results from the two approaches are superposed. It became evident that the repeated hold-out evaluation method generates more outliers than the other method (diamonds on Figure 5). It can be assumed that this difference is due to the training and validation generation processes since it is the only difference between the two given approaches.

The average RMSE on the test set is 0.099 for the repeated cross-validation procedure and 0.104 for the repeated holdout evaluation procedure. With a mean flux of 83.461, the RMSE represents 0.12% of error for the average flux in both methodologies.

**Figure 6:**
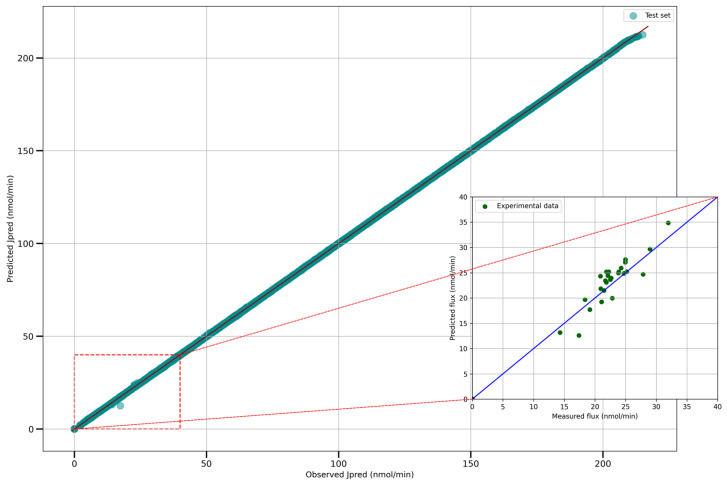
Comparison between expected and predicted output flux on the test set (the five test sets are concatenated into one). Find in the bottom right rectangle the difference between the expected and predicted output flux on the experimental data.

Moreno-Sánchez et al. [39] propose a set of experimental wet lab data for this metabolic pathway. Find in Table S3 in Supplementary Information the experimental set with its 29 instances. In our study, this experimental set is mixed with the training set to enhance the model’s performance. Evaluating the model on the experimental data is a good way to validate the model. In addition, a new DNN model is trained with the same hyperparameters after removing the 29 experimental wet lab instances from the training set to avoid data leakage. In the mainframe of Figure 6 below, there is a comparison between the predicted and expected value of the output flux based on the instances from the test set. In the bottom right rectangle, there is the same comparison, but this time with the experimental wet lab set alone. Performances on the experimental wet lab data (RMSE: 2.0412, R^2^: 92.9482%, MAE: 1.6457) are not as good as those obtained on the test set used in our study. Nevertheless, it allows us to validate the DNN model used in this study.

In the study of Lo-Thong et al. (2022) [30], they compared 13 different ML approaches, and the good performance of the Cubist model was noted. In their work, a simpler ANN was tested and outclassed by the Cubist. To assert our DNN model performance, two ML models are used as baselines: a non-linear approach, Cubist, and a linear one, Partial Least Squares regression (PLS), to illustrate the need for a non-linear model. The PLS model reaches an average of 30.663 on RMSE and 0.989 on R^2^, whereas the cubist model has a mean RMSE of 0.096 and R^2^ of 0.999 on the five test sets. In our case, by selecting the best DNN model based on the validation set in each procedure, we obtain an RMSE ranging from 0.069 to 0.123 on the five test sets with cross-validation and from 0.067 to 0.121 with repeated holdout evaluation. Table 6 shows RMSE results of DNN models and baseline models on the validation set and the average on all test sets. While the PLS model performs very poorly in comparison to the other three, the DNN models obtained from repeated cross-validation and repeated hold-out evaluation perform a bit better than the Cubist model with a slightly lower RMSE (≤0.01). With almost similar performance, the required computing time to train our DNN model and Cubist tends to point to the latest as a more convenient option. However, as stated by Kübler et al. (2021) [40], with the no free lunch theorem, it is not possible to know in advance without prospecting which learning algorithm will perform better than the other ones. The only solution to identify an ML model performing better than a DNN model is to train and test each one, which can outlast a rough hyperparameter tuning and training process of a decent DNN. In other cases, with large-scale datasets, more complex data, or unstructured data, DL models could automatically detect the best features and significantly outperform any ML algorithm, including a Cubist model [41,42]. The inference time (i.e., the time needed for the model to process all given information) can also be a criterion to choose a DNN model over a Cubist one. With less than a second to process the entire test set (<0.35 s), the DNN model is significantly faster than the Cubist model that took 17.523 s to process the same amount of information.

The development of the two tools REKINDLE [26] and RENAISSANCE [27] demonstrates impressive capabilities of deep learning techniques in generating kinetic models and parameterizing metabolic networks while reducing parameter uncertainty. However, a notable drawback of these studies is their focus on well-studied model organisms, such as *Escherichia coli*, for which abundant and rich datasets are available. This reliance on dense datasets limits the generalizability of these approaches to other less-characterized organisms or more complex biological systems with sparse data.

A common limitation across these studies is the challenge of validating DL model predictions with robust experimental data. While these approaches exhibit high performance on synthetic or predefined datasets, their ability to adapt to dynamic biological environments or unforeseen scenarios remains uncertain. These studies underscore the importance of diversifying model organisms and strengthening experimental validation to ensure the relevance and robustness of the results.

In our study, the results are validated against the limited experimental data available, and while this provides confidence in our models, we acknowledge the need to generate additional experimental datasets to further enrich and refine our models for broader applicability. Moreover, the metabolic system tested is relatively simple. It would be interesting to test these approaches on more complex metabolic pathways such as the whole glycolysis pathway consisting of 10 steps, the Benson Calvin cycle with 13 enzymatic reactions [43], or on the tryparedoxin-dependent hydroperoxide detoxification pathway in *Trypanosoma cruzi* [44].

The work of S. van den Bogaard et al. [45] makes a significant contribution by proposing sEnz, which enables sensitive enzymes to be identified and sensitivity coefficients to be calculated. Their approach provides a detailed analysis of protein constraints influencing metabolic fluxes. However, this method relies heavily on the availability of precise kinetic and proteomic parameters, which may limit its applicability. Our work, on the other hand, exploits advances in deep learning to predict metabolic fluxes from variations in enzyme concentrations, without requiring exhaustive knowledge of kinetic parameters. This approach is particularly useful in contexts where experimental data are limited, offering a robust alternative for modeling less well-defined biological systems. The two approaches could eventually complement each other: sEnz could highlight fundamental metabolic constraints, while our method would expand predictive possibilities for more diverse biological systems.

While the hyperparameters chosen to build the models provide interesting results, we cannot exclude that a model with different hyperparameters or with a different initialization of the initial parameters can offer better results. Optimizing hyperparameters by manual search of trial and error is time-consuming, but it can be automatized with Neural Architecture Search [46] (NAS), for example. Another limit is that in the field of DL, a dataset of 68,950 entries with only three features is considered a small dataset; enriching the dataset with diverse enzyme concentrations/flux couples might improve the performance of the models. In fact, in many metabolic pathway optimization studies, acquiring more entries and features is not uncommon thanks to omics technologies.

Inaccuracy in predicted metabolic flux values can impact industrial production processes: It is therefore important to be able to predict flux values as accurately as possible to correctly estimate the production yield of a process and the time required to obtain the desired quantity of target molecules. Furthermore, the approaches described in this paper may also be of interest for modeling non-linear cellular or acellular production systems.

This study elucidates the pioneering role of DL in metabolic pathway modeling and underscores its synergistic interplay with knowledge-based approaches and wet lab experiments. While DL models stand out for their adeptness at rapid data processing, high scalability, and predictive accuracy, knowledge-based models contribute substantially thanks to their structured, rule-based insights, enabling a more thorough and nuanced understanding of metabolic processes. The economic benefits of DL models are substantial, providing substantial savings in resources and time compared to the intensive demands of wet lab experiments.

The interplay of DL and knowledge-based models paves the way for groundbreaking advances in biotechnology research by combining the strengths of data-driven insights with rule-based reasoning. This symbiotic relationship can not only accelerate the discovery process but also ensure a more reliable and comprehensive exploration of metabolic pathways, optimizing both innovative potential and resource allocation in pharmaceutical and biochemical research endeavors.

## 4. Materials and Methods

### 4.1. Materials

#### 4.1.1. Dataset Description

The authors of a previous study generously supplied the initial metabolic network of the glycolysis pathway [39]. Even though this model is created using an older software (GEPASI version 3.21, https://gepasi.software.informer.com/, accessed on 5 February 2024), we use its successor COPASI (COmplex PAthway Simulator, version 4.42) to study the network (https://github.com/copasi/COPASI/releases, accessed on 5 February 2024).

The low part of the glycolysis pathway is developed in one of our previous studies [20] using the same metabolic pathway modeling software. This open-source software involves designing, analyzing, and optimizing metabolic networks, which are constructed using enzyme properties such as kinetic parameters and mechanism-based rate equations. The model was validated using experimental measurements adopted in a procedure described hereafter. We extracted 68,950 experimental conditions, which included a mixture of the enzymes 3-phosphoglycerate mutase (PGAM), enolase (ENO), and pyruvate phosphate dikinase (PPDK), as well as pathway flux (Jobs), using this model of the metabolic pathway. Data generated using a knowledge-based model are shown in Table S1 (Training set) and Table S2 (Test sets 1 to 5) found in the GitHub repository.

The training set is composed of 55,160 instances (80% of 68,950), and each test set (1 to 5) is composed of 2,758 instances for a total of 13,790 instances (20% of 68,950). The training set and test set are mutually exclusive; there are no instances present in both sets.

#### 4.1.2. Experimental Flux Measurements

The lower part of the glycolysis pathway has been reconstituted in vitro using recombinant enzymes. The reactions are made to progress under conditions found in the parasite *Entamoeba histolytica*. Once the reactions have reached a pseudo-steady state, samples are taken at two distinct time points. These samples are then processed for metabolite determination, including 2-phosphoglycerate (2PG) and phosphoenolpyruvate (PEP). This step allows for the quantification of metabolites generated by enzymatic reactions, providing a precise insight into the metabolic flux within the studied system. The entire procedure is repeated multiple times, varying the concentration of a single enzyme while keeping the others at constant values. Therefore, while enzyme concentrations will be integrated as an input into the models built, flux measurements will be the goal of predictions. For further details on the methodology employed, refer to the article by R. Moreno-Sanchez et al. (2008) [39].

#### 4.1.3. Artificial Neural Networks and Deep Neural Networks

Artificial neural networks are inspired by the neurons of the human brain; their structure makes it possible to retrieve information and process it before returning an output. This is achieved by a succession of three layers: input layer, hidden layer, and output layer. The “hidden layer” can itself be composed of several layers of neurons. When the neural network becomes deeper, with several layers, we can call it a “Deep Neural Network” (DNN). This neural network architecture is capable of handling classification or regression problems. This work was conducted with the Python programming language (version 3.8) using the Jupyter Notebook application (open source) as well as several libraries, including TensorFlow 2.0 for the use of neural networks.

The DNN model used in this study is composed of three hidden layers of 105 neurons each with ELU as an activation function. With a single neuron in the output layer, the total number of trainable parameters reaches 22,786. MSE is the chosen loss function with Adam to optimize parameters. The model is trained for 3000 epochs with a batch size of 100. An EarlyStopping function monitors the RMSE on the validation set with a patience of 100 epochs to avoid overfitting. Find in SI the hyperparameter tuning process used in this study.

### 4.2. Methods

#### 4.2.1. Dataset Analysis

**Figure 7:**
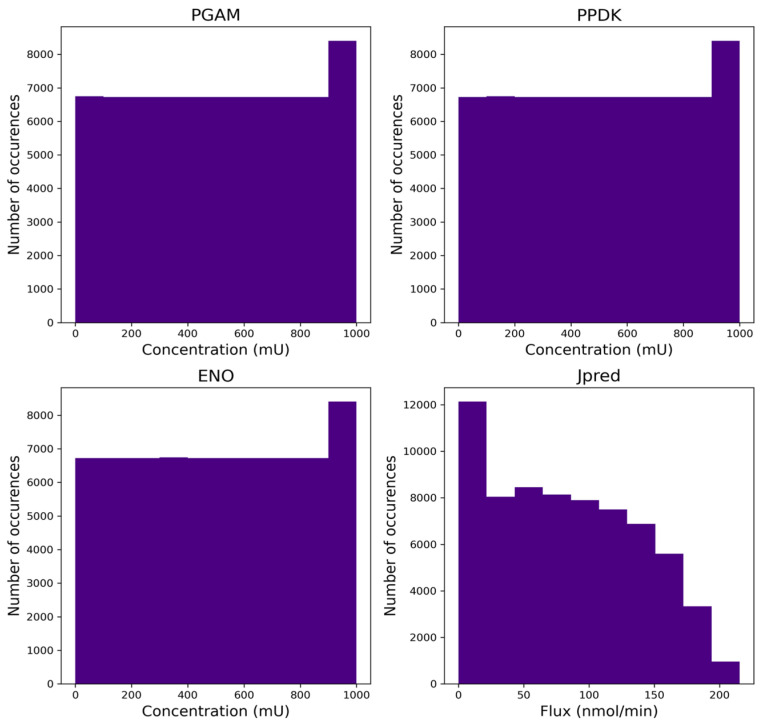
Distribution of the different variables in the Glycolysis database. Find in this figure, the number of occurrences of concentration values for the features (PGAM, ENO, and PPDK) and flux values for the target (Jpred). The variables PGAM, ENO, and PPDK lie in a uniform distribution. The variable J_pred_, corresponding to the output of the model, seems to follow a gamma distribution.

The Glycolysis database used in this study contains 68,950 entries with 4 variables. PGAM, ENO, and PPDK are the concentrations of the enzymes conditioning the production system, and J_pred_ is the output flux. The model should be able to determine J_pred_. from the variables PGAM, ENO, and PPDK. Figure 7 shows the distribution of the different variables in the database. Find additional information about the dataset in Table 7 below.

According to the Pearson correlation coefficient [47], the PGAM enzyme has the greatest impact on the J_pred_ output flux with a value of 0.63. The other 2 variables, ENO and PPDK, have a more moderate impact with values of 0.49 and 0.26, respectively. The 3 variables PGAM, ENO, and PPDK are not correlated with each other.

#### 4.2.2. Baseline Workflow to Process Data: Holdout Evaluation

Before starting to process the data into the model, it is necessary to normalize the training and test set. Normalization of the training set will speed up the convergence of the model. Not only are the features normalized (PGAM, ENO, PPDK), but also the value to predict J_pred_. To make the results more readable, we de-normalized the expected values and predicted values before computing the metrics. Since the model is trained with normalized data, the test set needs to be normalized for the model to work properly. In this study, MinMax normalization has been chosen:

(1) $$z_{i}=\frac{x_{i}-min(x)}{max\left(x\right)-min(x)},$$

**Figure 8:**
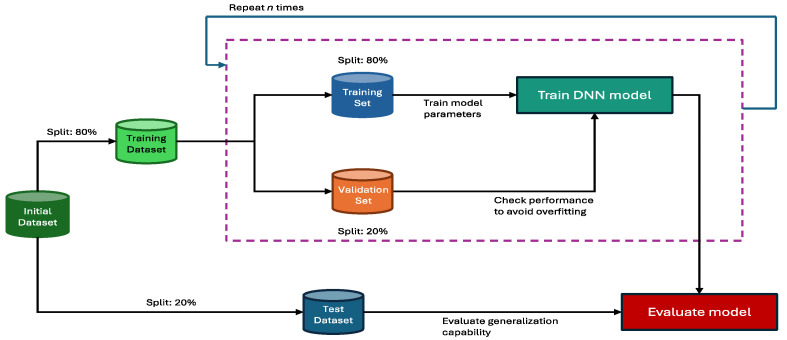
Holdout evaluation workflow for a deep learning model. First, the initial dataset is separated into two datasets: a training set and a test set. Given the size of our dataset, a random separation of 80/20 generally allows us to have both two sub-datasets respecting the distribution of the initial dataset and a test set with a satisfactory size. Then, the training set is split in two (80/20) to obtain a validation set in addition to the training set. The remaining training set will be used to train the model, and the validation set will be used to check if the model is overfitting. Once the training is completed, the model is evaluated on the test set.

From the training dataset, a training set and validation set are created using an 80/20 split. The training set is used to optimize the model’s parameters, and at each epoch, the model is only evaluated on the validation set. This allows us to monitor if the model is overfitting or not. When the training is completed, the model is evaluated on the test dataset initially set aside to assess its generalization capabilities (see Figure 8).

#### 4.2.3. First Procedure: Repeated Cross-Validation

**Figure 9:**
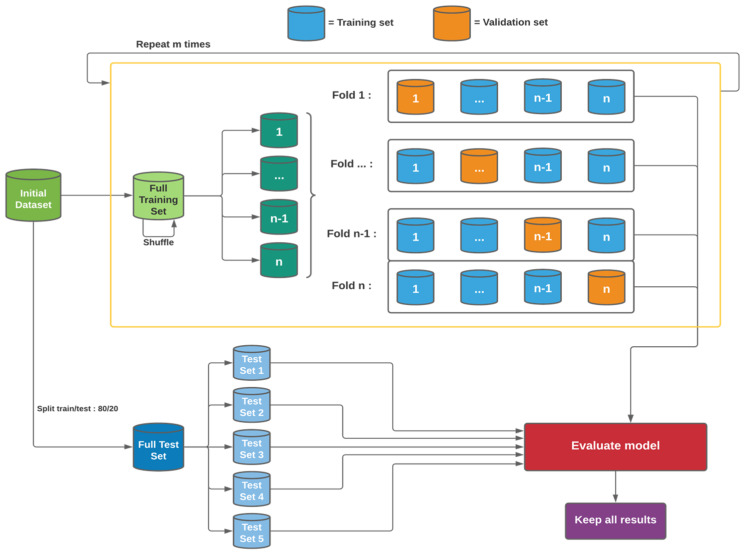
Workflow of the repeated cross-validation approach. First, the initial dataset is separated into 2 data subsets: one set to train the models and the other to evaluate them. The test dataset is separated into 5 test datasets in order to test the generalization capabilities of the models. In the large yellow box, the training data is separated into several folds. One fold will act as a validation set and the others as a training set when training a model. To complete a repetition, each fold will act as a validation set one time. This process will be repeated m times. All the generated models are evaluated on the test sets in order to collect all the performances of each model.

As in the baseline workflow for an ML model, to avoid any risk of data leakage, where the data used to assess the generalization performance can end up in the training process, a test set is created and set aside from the training set (see Figure 9). The test set is divided into five subsets to better test the generalization capabilities of the models and because the size of the dataset allows it. Since each model is tested on the same data, it is therefore possible to directly compare the results obtained. As the cross-validation workflow defines it, the remaining training set is split into 5 with respect to the distribution of the initial dataset.

During the training process, one of the five folds serves as a validation set while the other four serve as a training set. The model’s performance is conditioned by its results on the validation set, where the training set is used to optimize the model weights and the validation set to stop the training at the peak performance on unseen data. Then, to evaluate the generalization capabilities of the model, the test sets are forwarded in the model to compute different metrics. This is repeated four times until each fold has served as a validation set. This whole process is repeated 10 times, except the test sets that remain the same, so that each model is evaluated on the same data set aside initially to have a fair comparison of generalization performance.

#### 4.2.4. Second Procedure: Repeated Holdout Evaluation

The workflow for this procedure is a mix of the hold-out evaluation in Figure 8 and the repeated cross-validation in Figure 9. While the cross-validation method can be viewed as robust for a small dataset by testing if each subset has the same impact on model performances, holdout evaluation relies on randomness to generate the training and validation set and is less “risky” to use on a large-scale dataset. In the present study, testing this evaluation method served to determine if our dataset was big enough for this approach to generate models with performances equivalent to those generated by cross-validation.

In this approach, to test the generalization capabilities of each model, the workflow follows the one introduced in Figure 9, from the repeated cross-validation. The test set is split into 5 subsets as the dataset size allows it. To generate the training and validation sets, it follows the upper part of Figure 8, the baseline workflow. 20% of the training is used as a validation set, and the remaining 80% is used to optimize the model’s parameters. Once the training is over, the model is evaluated on the 5 test sets. This process is then repeated 50 times, and at each new iteration, the training set and validation set are mixed and shuffled to generate new random training and validation sets. The repetition is highlighted in Figure 8 by the purple dotted block. The test sets stay the same to allow a fair performance comparison.

#### 4.2.5. Computational Time

A last element of comparison between these two procedures is the computation time required for their execution. In our work, where we generated and trained the same number of models with both methods, the computation time is roughly the same. These experiments were performed using an Intel^®^ Xeon^®^ Gold 5218R @ 2.10 GHz on a server of DSIMB laboratory at University of Reunion (Saint-Denis, France) and each procedure took approximately 11 h.

#### 4.2.6. Correlation Metrics to Compare Performance

In each procedure described above, results are obtained on 5 test sets. Here are 3 statistical tools used in this study to assess whether, if a model performs better on a test set, it also performs better on the other four test sets: Kendall’s tau [37], Spearman’s rank correlation [38], and Pearson’s correlation coefficient.

- Kendall’s tau: When taking pairwise results, the number of matching pairs as well as the number of mismatched pairs should be used to measure the rank correlation between the test sets. Let $\left(x_{1},y_{1}\right),\left(x_{2},y_{2}\right),\ldots ,\;\left(x_{n},y_{n}\right)$ be a set of results where X = ($x_{1},x_{2},\ldots ,x_{n}$) corresponds to a test set and Y = ($y_{1},y_{2},\ldots ,y_{n}$) to the other. A pair of results $\left(x_{i},y_{i}\right),\;\left(x_{j},y_{j}\right)$ will be said to be concordant if $x_{i}<x_{j}\;and\;y_{i}<y_{j}$ or if $x_{i}>x_{j}\;and\;y_{i}>y_{j}$. Otherwise, the pair will be discordant.

(2) $$\tau =\frac{\left(number\;of\;concordant\;pairs\right)-\left(number\;of\;discordant\;pairs\right)}{\frac{n\left(n-1\right)}{2}},$$

with $\frac{n\left(n-1\right)}{2}$ = total number of pairs.

- Spearman’s rank correlation: After sorting the data in ascending order, we replace the results by their rank:

(3) $$r_{s}=\frac{cov({rg}_{X},{rg}_{Y})}{\sigma_{{rg}_{X}}\sigma_{{rg}_{Y}}},$$

where X corresponds to a set of results on one test set and Y on the other test set; $rgX_{i}$ and $rgY_{i}$ are the rank variables calculated from the data $X_{i}$, $Y_{i}$; $cov({rg}_{X},{rg}_{Y})$ is the covariance of variables of rank; $\sigma_{{rg}_{X}}$ and $\sigma_{{rg}_{Y}}$ are the standard deviations of rank variables.

- Pearson’s correlation coefficient:

(4) $$\rho_{X,Y}=\frac{cov\left(X,Y\right)}{\sigma_{X}\sigma_{Y}},$$

with X corresponding to the results of one set of tests and Y to the other; cov is the covariance, $\sigma_{X}$ and $\sigma_{Y}$ are the standard deviations of X and Y.

## Figures and Tables

**Table 1:** Summary of every hyperparameter chosen for the DNN model.

Hyperparameter	DNN Model
Number of hidden layers and neurons per layer	3 hidden layers 105/105/105
Activation function in hidden layer	ELU
Number of neurons in the output layer	1
Activation function in output layer	Sigmoid
Trainable parameters	22,786
Loss function	MSE
Optimizer	Adam
Number of epochs	3000
Batch size	100
Metrics	RMSE, R^2^, MAE
EarlyStopping monitoring metrics with patience	Validation RMSE, with a patience of 100 epochs

**Table 2:** Model results on the training and validation data at epoch 794 as well as results on the test set after training. The MSE, RMSE, R^2^, and MAE metrics allow us to appreciate the performance of the model. The results on the validation and test data are very close. The model generalizes well.

Metrics	Train Set	Validation Set	Test Set
MSE	0.006	0.006	0.007
RMSE	0.077	0.081	0.083
R^2^	0.999	0.999	0.999
MAE	0.051	0.051	0.051

**Table 3:** Metrics performance of all 50 models on the validation set and the five test sets using repeated cross-validation. For each metric, the mean performance on the validation set is close to those on each test set, assessing the good generalization capability of models.

Sets	Validation Set	Test Set 1	Test Set 2	Test Set 3	Test Set 4	Test Set 5
RMSE	[0.081, 0.115]	[0.069, 0.099]	[0.117, 0.148]	[0.078, 0.113]	[0.074, 0.106]	[0.074, 0.106]
	Mean: 0.096	Mean: 0.084	Mean: 0.133	Mean: 0.096	Mean: 0.088	Mean: 0.088
R^2^	[0.999, 0.999]	[0.999, 0.999]	[0.999, 0.999]	[0.999, 0.999]	[0.999, 0.999]	[0.999, 0.999]
	Mean: 0.999	Mean: 0.999	Mean: 0.999	Mean: 0.999	Mean: 0.999	Mean: 0.999
MAE	[0.052, 0.079]	[0.051, 0.077]	[0.055, 0.082]	[0.051, 0.078]	[0.053, 0.079]	[0.053, 0.079]
	Mean: 0.063	Mean: 0.061	Mean: 0.066	Mean: 0.063	Mean: 0.062	Mean: 0.062

**Table 4:** Results on all the test sets for models generated and trained using repeated holdout evaluation. As in the cross-validation approach, the models have a good generalization capability.

Sets	Validation Set	Test Set 1	Test Set 2	Test Set 3	Test Set 4	Test Set 5
RMSE	[0.072, 0.146]	[0.067, 0.135]	[0.121, 0.174]	[0.075, 0.151]	[0.072, 0.136]	[0.072, 0.142]
	Mean: 0.101	Mean: 0.089	Mean: 0.134	Mean: 0.098	Mean: 0.093	Mean: 0.093
R^2^	[0.999, 0.999]	[0.999, 0.999]	[0.999, 0.999]	[0.999, 0.999]	[0.999, 0.999]	[0.999, 0.999]
	Mean: 0.999	Mean: 0.999	Mean: 0.999	Mean: 0.999	Mean: 0.999	Mean: 0.999
MAE	[0.051, 0.095]	[0.049, 0.095]	[0.054, 0.099]	[0.051, 0.097]	[0.049, 0.096]	[0.052, 0.096]
	Mean: 0.066	Mean: 0.065	Mean: 0.069	Mean: 0.066	Mean: 0.066	Mean: 0.066

**Table 5:** *t*-test values comparing the results obtained with the two approaches presented for each metric. This statistical test tool indicates whether or not it is possible to reject the hypothesis that the data of the two approaches have the same square error mean and absolute error mean. Depending on the test sets, the *p*-values are either higher than 0.05 (test sets 1, 2, and 3) or lower (test sets 4 and 5).

*p*-Value	Test Set 1	Test Set 2	Test Set 3	Test Set 4	Test Set 5
RMSE	0.095	0.486	0.469	0.029	0.053
R^2^	0.069	0.442	0.335	0.023	0.043
MAE	0.059	0.116	0.107	0.034	0.011

**Table 6:** RMSE results comparison between baseline ML models and the best DNN models according to performance on the validation set. Results on the five test sets have been averaged with the standard deviation.

Model	Validation Set	Test Set
PLS Baseline	30.422	30.663±0.46
Cubist Baseline	0.104	0.096±0.01
DNN: Repeated cross-validation	0.081	0.086±0.02
DNN: Repeated holdout evaluation	0.072	0.084±0.02

**Table 7:** Synthetic table characterizing the information of the four variables in our dataset: PGAM, ENO, PPDK (enzyme concentration), and J_pred_ (output flux).

	Mean	Standard Deviation	Minimum	Maximum
PGAM	499.819	295.875	0	1000
ENO	499.916	295.778	0	1000
PPDK	499.865	295.818	0	1000
J_pred_	83.461	55.317	0	215.448

## Data Availability

The original data presented in the study and the Python code are openly available in the GitHub repository at https://github.com/freddy-oulia/Metabolic_fluxes_DNN.git (accessed on 6 October 2024).

## Supplementary Materials

The following supporting information can be downloaded at: https://www.mdpi.com/article/10.3390/ijms252413390/s1. References [48,49,50,51,52,53,54,55,56] are cited in the Supplementary Material.
