# Supplementary material for: Metabolic Fluxes Using Deep Learning Based on Enzyme Variations: Application to Glycolysis in Entamoeba histolytica
Source: Int J Mol Sci. 2024 Dec 13;25(24):13390. doi: 10.3390/ijms252413390 (PMC11676880; doi:10.3390/ijms252413390)
Supplement: Supplementary file 1 [file ijms-25-13390-s001.zip › ijms-3272619-supplementary.pdf]

Supplementary Information

MODELING METABOLIC FLUXES USING DEEP LEARNING BASED ON ENZYME VARIATIONS. *Application to glycolysis in Entamoeba Histolytica*.

Freddy Oulia[1,2, 3], Philippe Charton[1,2, 3], Ophélie Lo-Thong-Viramoutou [1,2, 3], Carlos G. Acevedo-Rocha[4], Wei Liu[5], Du Huynh[5], Cédric Damour[6], Jingbo Wang[7] and Frederic Cadet,[1, 2, 3, 8*]

[1] BIGR, UMR_S1134 Inserm, University of Paris City, 75006 Paris, France
[2] Laboratory of Excellence GR-Ex, 75006 Paris, France,
[3] DSIMB, UMR_S1134 BIGR, Inserm, Faculty of Sciences and Technology, University of Reunion, 97744 Saint-Denis, France,
[4] The Novo Nordisk Foundation Center for Biosustainability, Technical University of Denmark, DK-2800 Kgs. Lyngby, Denmark
[5] Department of Computer Science and Software Engineering, School of Physics, Mathematics and Computing, The University of Western Australia, Perth 6009, Australia
[6] EnergyLab, EA 4079, Faculty of Sciences and Technology, University of La Reunion, Saint-Denis, France,
[7] Department of Physics, School of Physics, Mathematics and Computing, The University of Western Australia, Perth 6009, Australia
[8] Artificial Intelligence Department, PEACCEL, 75013 Paris, France,

[*]Corresponding author: frederic.cadet.run@gmail.com

1. Hyperparameter tuning for neural network.

The selection of hyperparameters is part of the process of creating a neural network. A hyperparameter is a parameter that determines the structure of the neural network (number of layers, number of neurons…) or the way the learning will be done (algorithm that performs the gradient descent, for example). They should not be confused with model parameters (neuron weights) which are quantities that evolve during the training of the neural network.

There are different methods to determine each hyperparameter, such as manual search by trial and error which might be time consuming, or algorithms like grid search[1] or random search[2]. Even with the help of these algorithms, this step remains time and resource consuming. The following are the hyperparameters that will be used for each model generated in this work:

- The loss function allows us to measure the performance of a model through a metric, a numerical value. This metric represents the error made by the model. To minimize this function and attain a better predictive capacity, the model will use this loss and correct its parameter. The Mean Squared Error (MSE) is commonly used as a loss function in Deep Learning models in regression problems. In our case, where the model has to predict the output flow of the Glycolysis metabolic pathway, which is a continuous value, this function fits our need.
- SGD[3], RMSprop[4], and Adam[5] are the most used algorithms to optimize the parameters of the neural network during backward propagation[6]. After several experiments with each algorithm on a Deep Neural Network (DNN) with 3 hidden layers (64/32/16), we chose the Adam optimizer because it converged faster and led to the best results.

Overfitting occurs when a model performs well on known data and performs poorly on never-seen data. A sign of overfitting is when, during the training process, a metric starts to improve on the training set and to deteriorate on the validation set. Another sign can be when the performance on the validation set is significantly better than on the test set. Overfitting leads to poor generalizability of the model. To mitigate the overfitting problem, we adopt the following strategies:

- EarlyStopping[7]. This is a mechanism that prevents the model from overfitting. During the training, this function monitors a chosen metric and if it does not improve during a defined number of epochs (the patience), the training is forcibly interrupted. The model parameters are then reloaded at the epoch where the model had the best results (according to the monitored metric). In our case, EarlyStopping monitors the RMSE on the validation set with a patience of 100 epochs.
- Setting the number of training epochs appropriately. There are different elements to consider when choosing a suitable number of epochs. It must be large enough to give the model sufficient time to converge but not too large, to avoid overfitting. Since EarlyStopping avoids overfitting, we can choose a sufficiently large number of epochs to allow the model time to converge. We chose 3000 epochs to train the models.
- Choosing suitable depth and breadth for the network. To determine the appropriate number of layers (depth) as well as the number of neurons per layer (breadth), it is necessary to conduct several experiments by varying these variables until satisfactory results are obtained (an RMSE on the validation set lower than a target value). We conducted a grid search with 2 and 3 hidden layers with a number of neurons per layer smaller than 150. The goal is to reach a model with a reasonably low complexity according to its number of parameters. We made sure that the number of parameters

in the DNN is smaller than the number of instances in the training set (55,160 instances). Table S1 shows a sample of results obtained for several experiments with each proposed structure. The best architecture obtained through grid search is a 3-layer neural network with 105 neurons in each hidden layer.

- Dropout. This is another technique that prevents overfitting[8]. However, during the experiments, it did not contribute to the improvement of performance and therefore it is not used in our model.
- Batch size. This is a hyper-parameter that helps to reduce the computational load by sending a batch of data instead of the whole set to the model in each training iteration. While a batch size of 1,000 generates results more quickly, a smaller batch size, such as 100, will generate more performant models.

Table S1. Experimental results to determine an efficient structure for the neural network architecture. The performances (RMSE, MAE and R2) of the models are evaluated on the validation set. The hyperparameters used during these experiments are: the MSE as loss function, ELU as activation function in all hidden layers, linear function in the output layer and Adam to optimize the parameters. The training lasted for 3000 epochs, but we implemented an EarlyStopping function to monitor the RMSE with a patience of 100 epochs. The best performance on the validation set is obtain with a 3-layer neural network (105/105/105) with a RMSE of 0.081.

Structure	Number parameters	RMSE	MAE	R2
2 hidden layers: 65/70 neurons	4,951	0.161	0.102	0.999
2 hidden layers: 85/90 neurons	8,171	0.178	0.111	0.999
3 hidden layers: 30/65/20 neurons	3,476	0.137	0.083	0.999
4 hidden layers: 64/86/100/124 neurons	27,195	0.084	0.053	0.999
5 hidden layers: 56/68/96/124/162 neurons	43,165	0.088	0.055	0.999
3 hidden layers: 105/105/105 neurons	22,786	0.081	0.051	0.999

The selected architecture for this study, as indicated in the last row of Table S1, features three hidden layers – 105 – 105 – 105. We chose the Exponential Linear Unit (ELU) function as the activation function in the experiments because it alleviates the vanishing gradient problem[9]. Sigmoid, tanh and ReLU are other widely used activation functions. Table 2 presents the results obtained using different activation functions. Figure S1 summarizes the DNN structure used during these experiments.

Table S2. Performance on validation set and training time when using different activation functions in hidden layers. The model is composed of 3 layers of 105 neurons each and the activation function in the output layer is the sigmoid function. To obtain comparable results, we removed the EarlyStopping function, and each model was trained for 1000 epochs. It is with the ELU activation function that we obtain the best model according to the RMSE. Each model was trained with Google Colab and their training times are similar.

Activation function	RMSE	Training time
ELU	0.1347	21 min 23
ReLU	0.1442	20 min 22
Sigmoid	0.1722	21 min 22
Tanh	0.5417	20 min 06

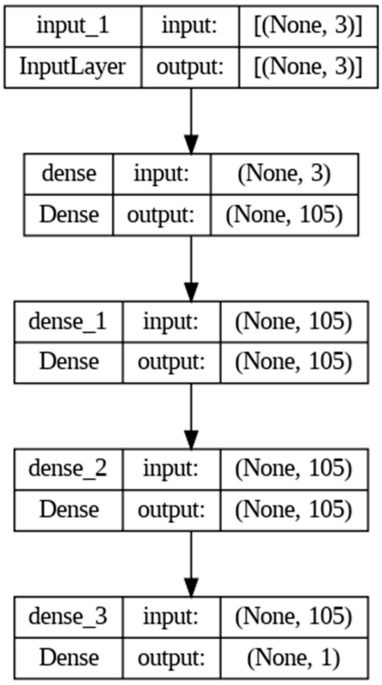

Figure S1. Architecture of the best neural network used in the paper (after cross validation). The DNN is composed of 3 hidden layers of 105 neurons. All these layers have ELU as activation function. The output layer is composed of a single neuron with the sigmoid activation function. The MSE has been chosen as a loss function and it is with the Adam algorithm that the model parameters are optimized. The maximum number of epochs has been set to 3000 and the training is done with EarlyStopping which monitors the RMSE on the validation data with a patience of 1000. The batch-size is 100. The performance of the model is evaluated using: RMSE, MAE and R2 metrics.

For Figure S2, the EarlyStopping function was removed, and we observe a slight improvement of RMSE on the validation set i.e., 0.069 instead of 0.081, after de-normalization, at the last epoch) and there is no sign of overfitting. Even the performance on the test set, with an RMSE of 0.071, does not suggest any sign of overfitting. The relative simplicity of both the model and the dataset could explain this.

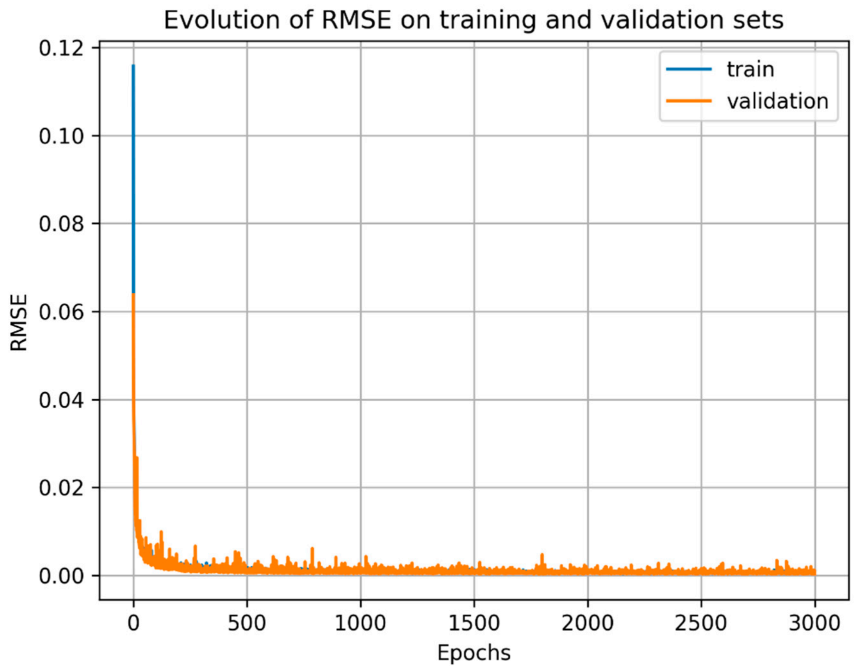

Figure S2. Evolution of RMSE on training and validation data of a model without EarlyStopping and with the number of epochs fixed at 3000. After the last epoch, the RMSE reached 0.069 on the validation set and 0.071 on the test set when we de-normalized the ground truth and prediction. There is no sign of overfitting.

The use of EarlyStopping has little impact on the performance but has significant impact on computation time. When using Google Colab with a Nvidia T4, the training for 894 epochs lasts about 22 min instead of 1 hour for the training of 3000 epochs. Not using the EarlyStopping function has only a marginal impact on performance since the difference in RMSE is about 0.02. Nevertheless, it multiplies the computation time by 3. As in each method, there are 50 models to be trained; we used the EarlyStopping function to save computation time. Although the main goal is to gain accuracy on the prediction of the output stream, it is appreciable that the computation time remains reasonable, especially if the performance improvement is not significant.

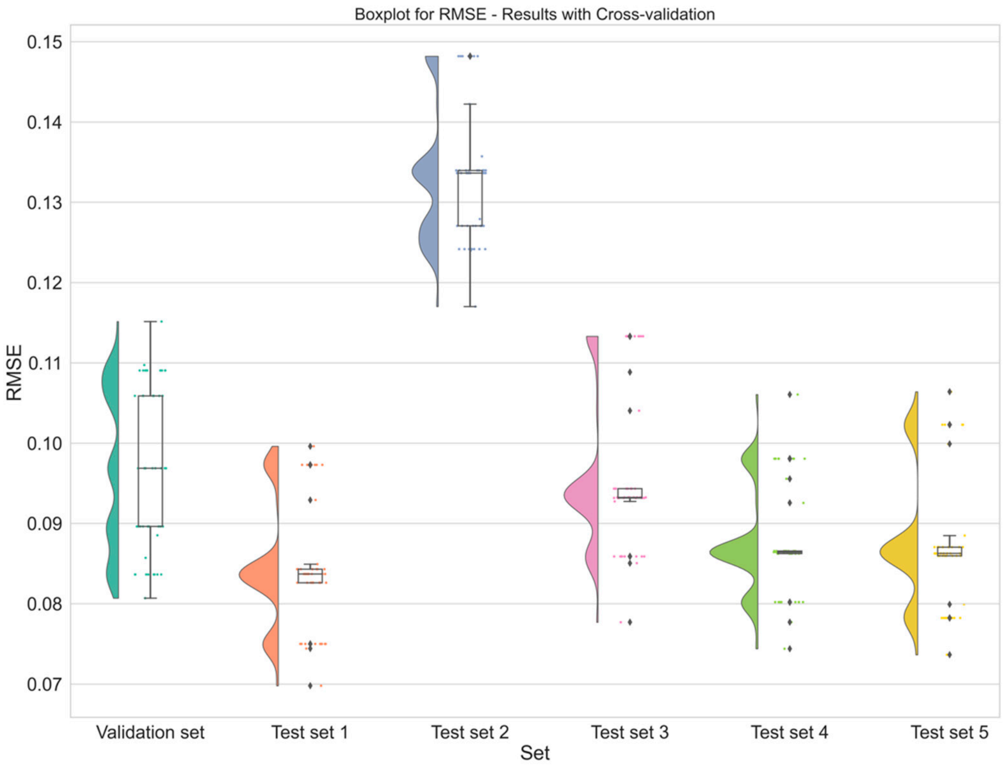

Figure S3. RMSE results using repeated cross-validation on the validation and the 5 test sets. For each plot, a boxplot with the density distribution is used to represent the RMSE value of each of the 50 trained models. The results obtained on the validation set and on the 5 test sets are comparable to an RMSE value between 0.07 and 0.15.

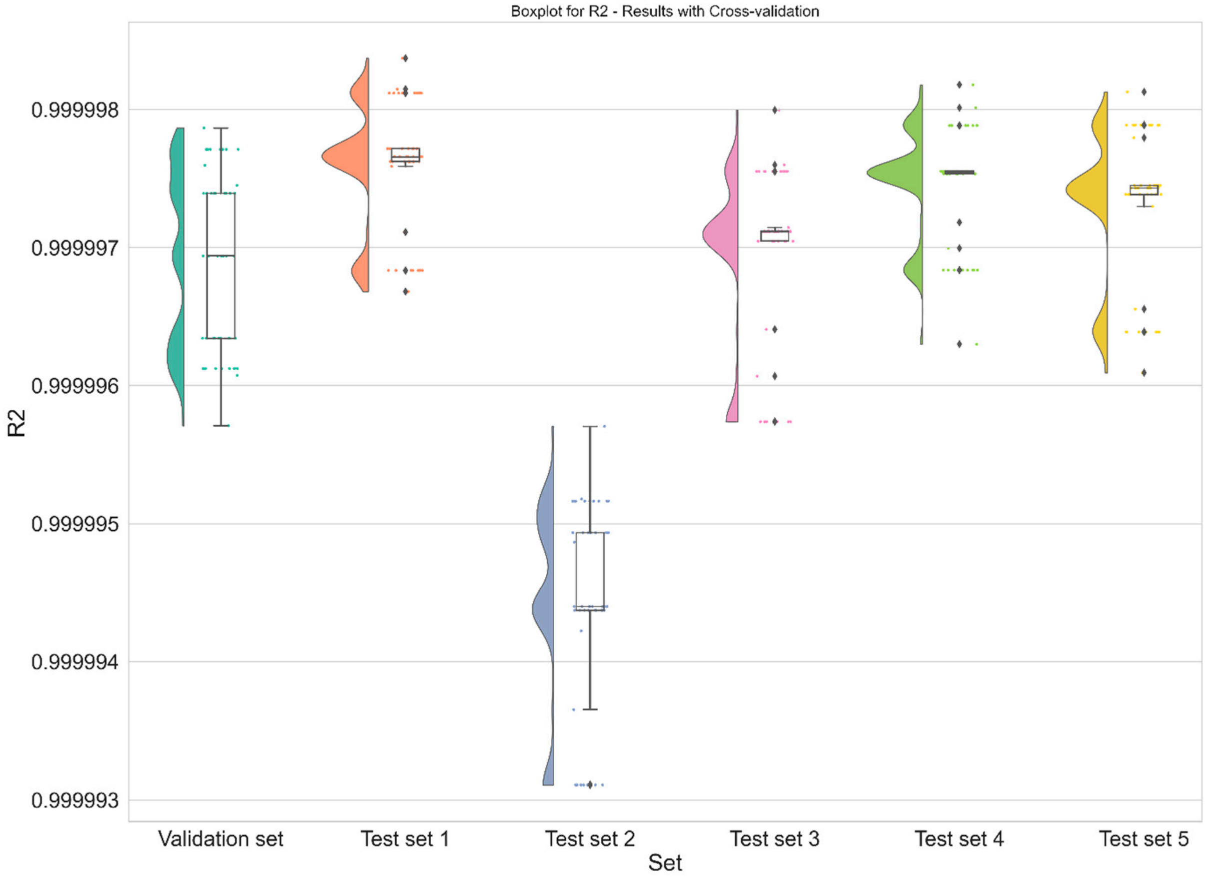

Figure S4: R^2 results using repeated cross-validation on the validation and the 5 test sets. For each plot, a boxplot with the density distribution is used to represent the R^2 value of each of the 50 trained models. The R^2 results obtained on the validation set and on the 5 test sets are higher than 0.999993.

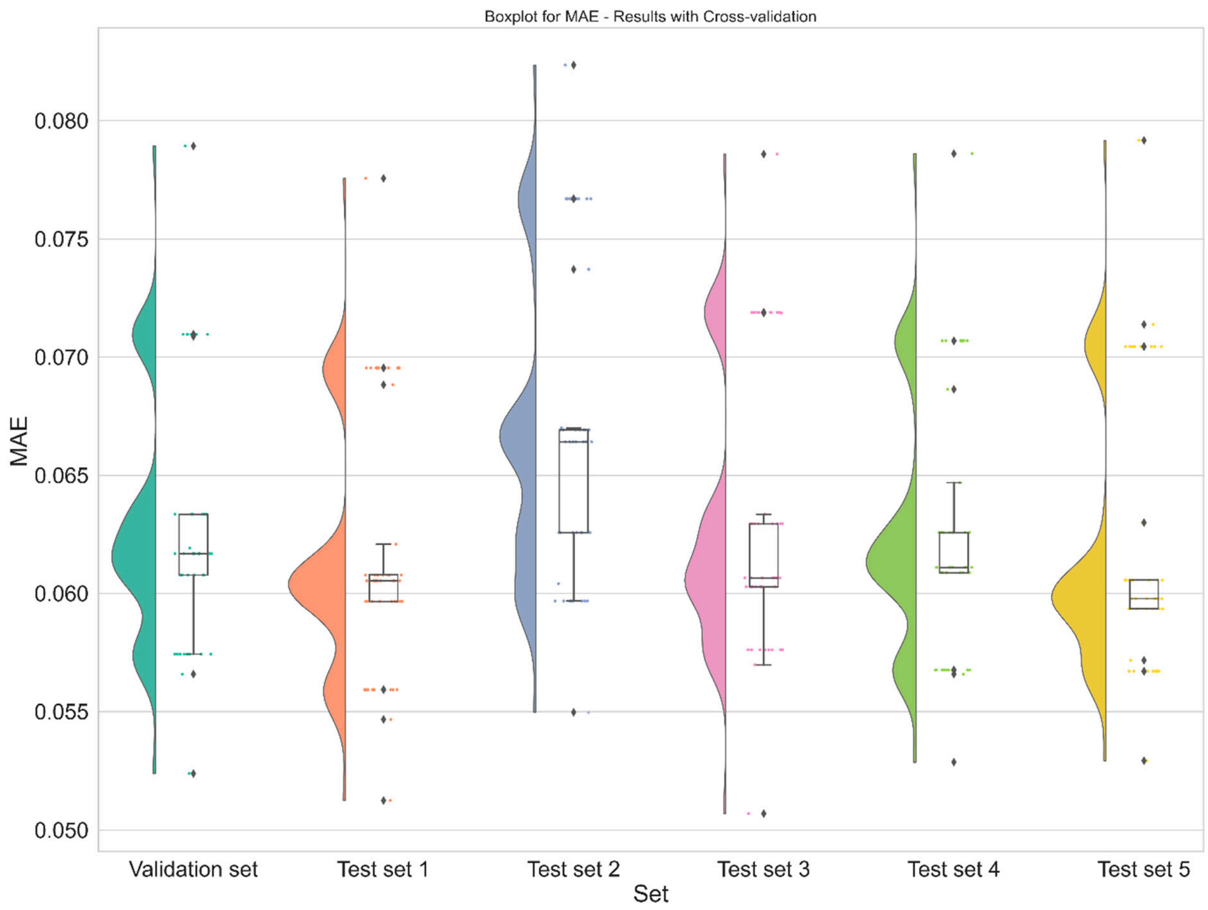

Figure S5: MAE results using repeated cross-validation on the validation and the 5 test sets. For each plot, a boxplot with the density distribution is used to represent the MAE value of each of the 50 trained models. The MAE results obtained on the validation set and on the 5 test sets range between 0.085 and 0.05.

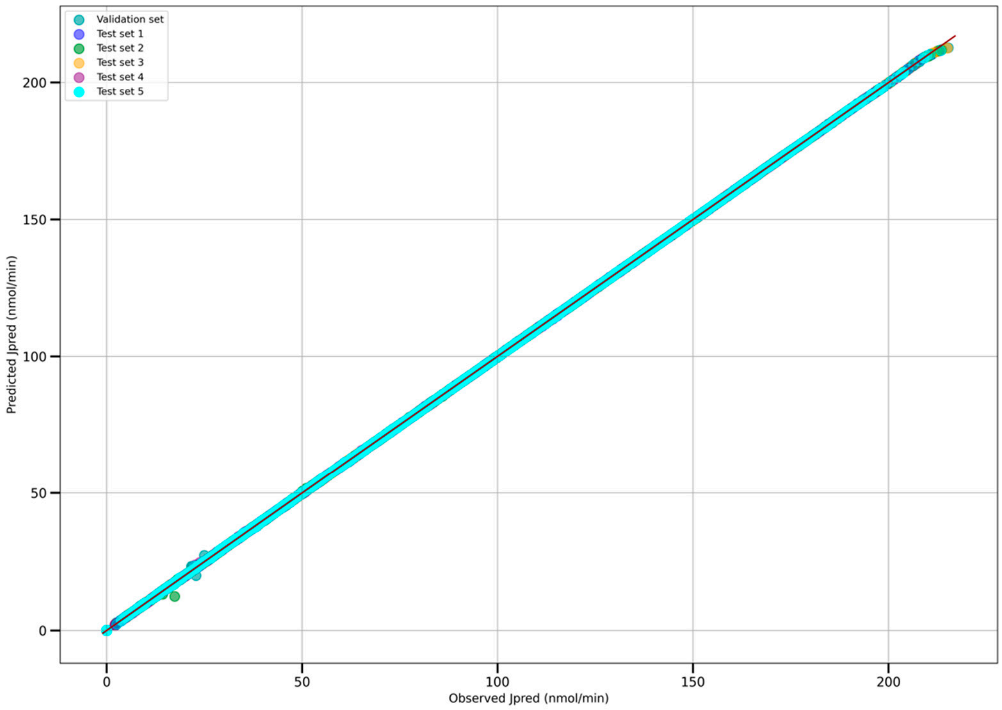

Figure S6: This plot shows the difference between the predicted and observed output flux J_{pred} from each instance in the validation set and the 5 test sets. These results are from a randomly selected model picked from the repeated cross-validation procedure. Instances from the different set overlap each other and apart for a few outliers, the predictions are almost perfect. This figure outlines the generalization capabilities of the model.

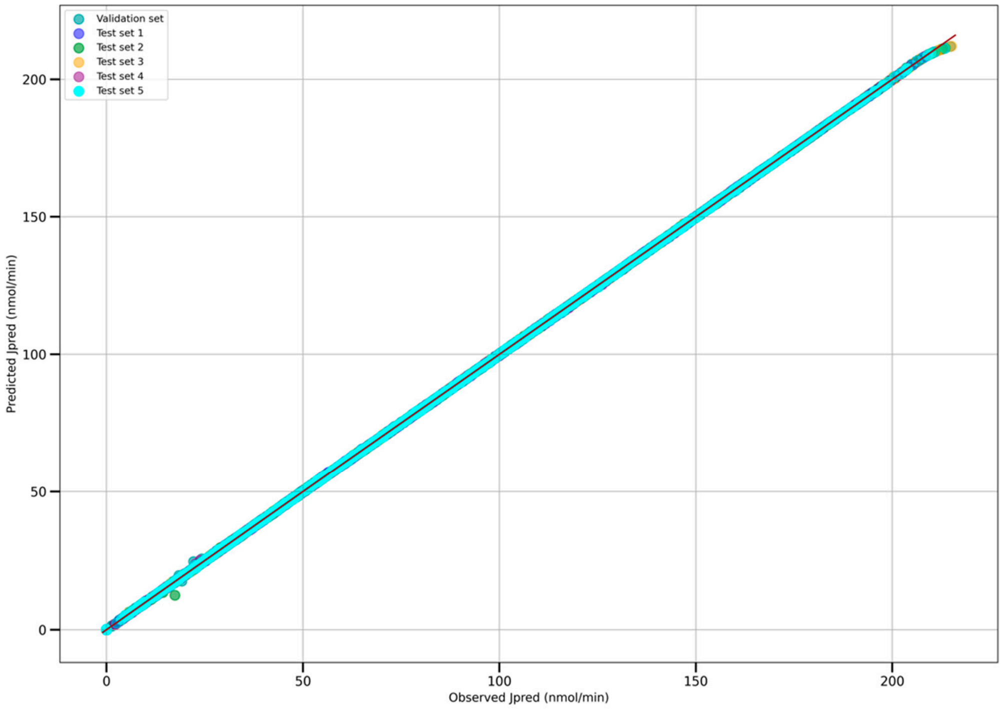

Figure S7: As Figure S6, this plot shows the difference between the predicted and observed output from the validation set and the test sets using another randomly picked model from the repeated cross-validation procedure. Despite using a different model, this figure is almost identical to the Figure S6 and any model among the 50 generated through cross-validation procedure reaches the same similarity. This empirically shows that each model has great generalization capacities.

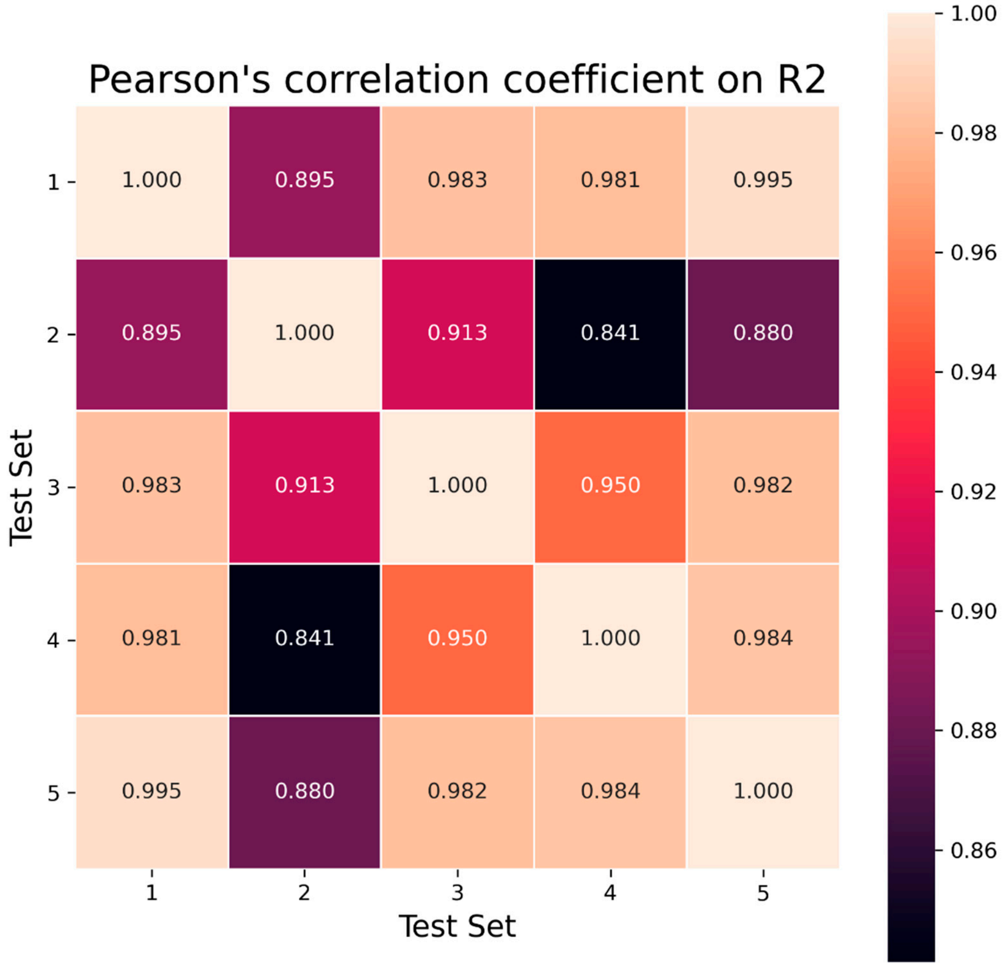

Figure S8: Heat map representing Pearson's correlation coefficient on the 5 test sets (numbered from 0 to 4) during repeated cross-validation. The correlation of the R^2 results between the different test sets is very strong (always higher than 0.8): The models have a good generalization capacity.

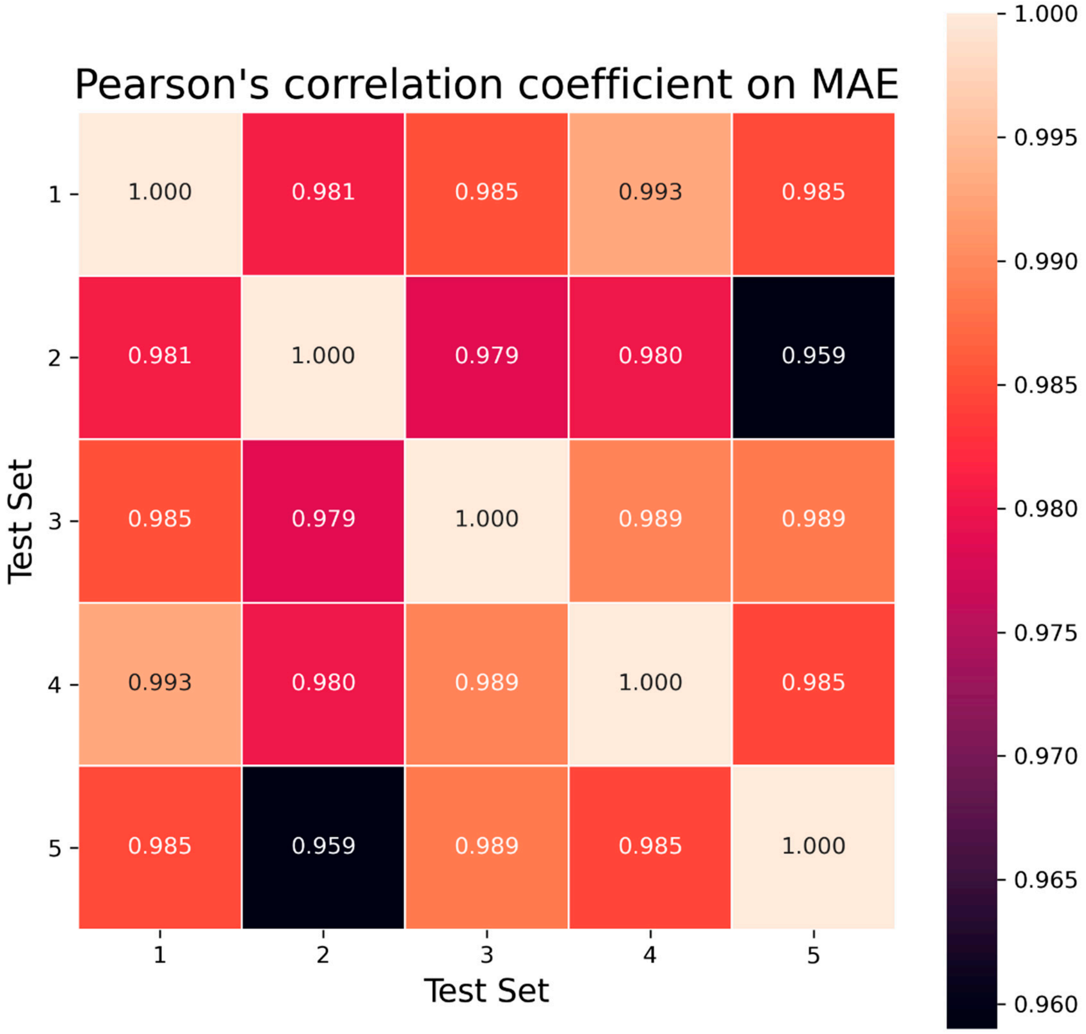

Figure S9: Heat map representing Pearson's correlation coefficient on the 5 test sets (numbered from 0 to 4) during repeated cross-validation. The correlation of the MAE results between the different test sets is very strong (always higher than 0.9): The models have a good generalization capacity.

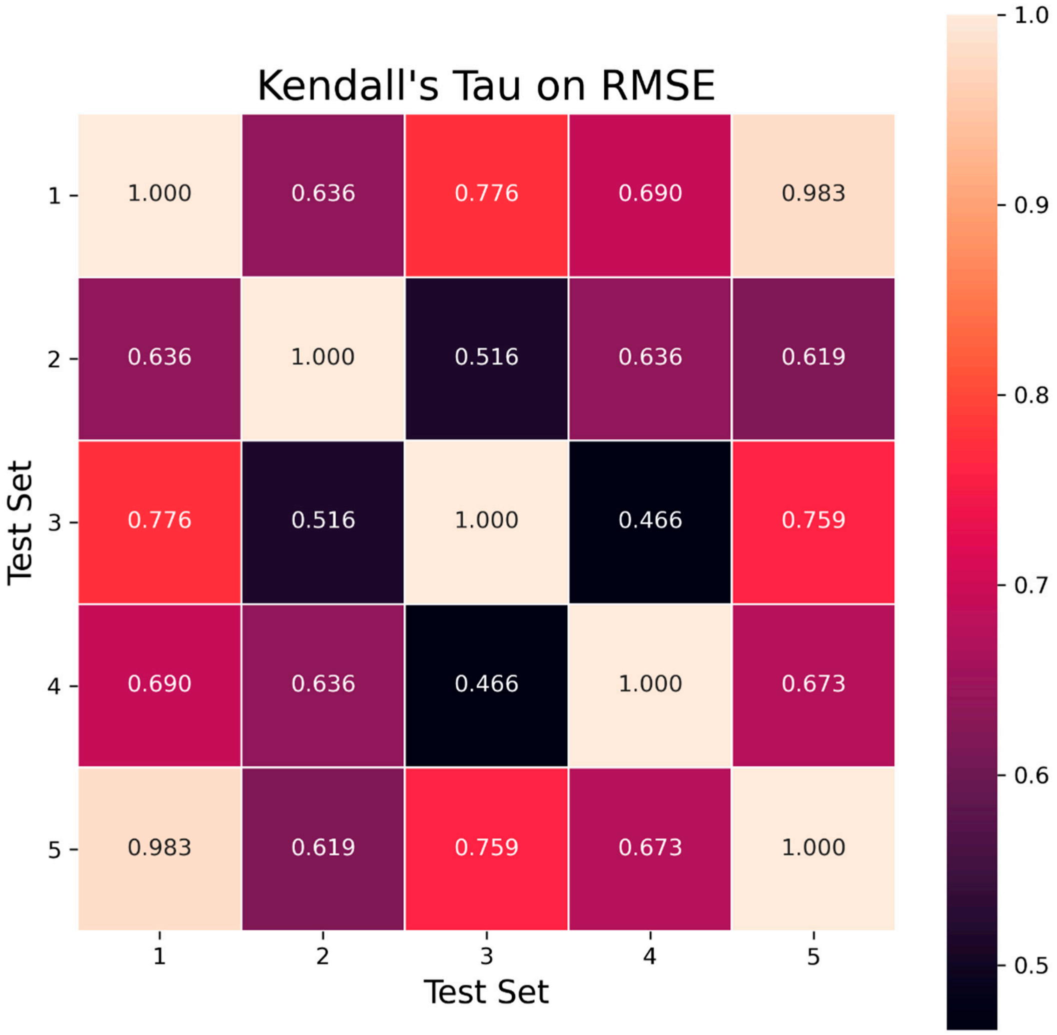

Figure S10: Heat map representing the Kendall's tau on the 5 test sets (numbered from 0 to 4) during repeated cross-validation. The correlation of the RMSE results between the different test sets range between 0.4 and 0.9.

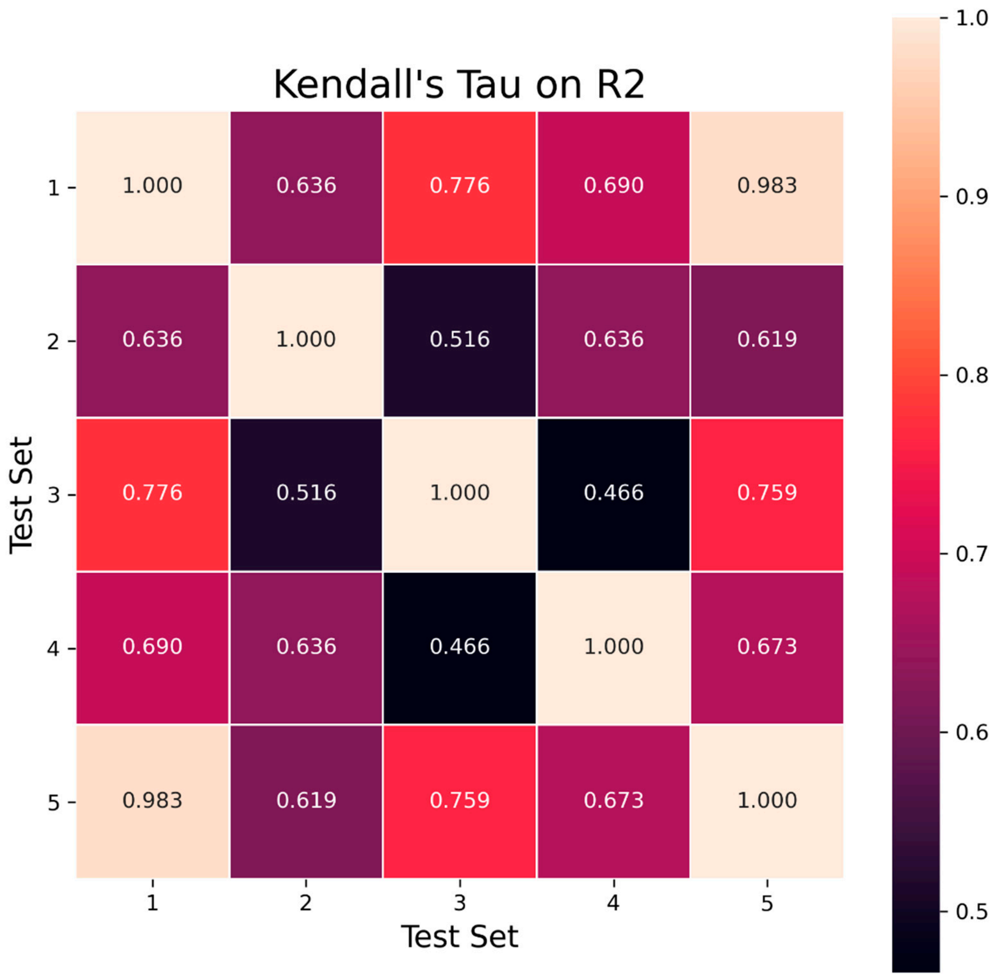

Figure S11: Heat map representing the Kendall's tau on the 5 test sets (numbered from 0 to 4) during repeated cross-validation. The correlation of the R^2 results between the different test sets range between 0.4 and 0.9.

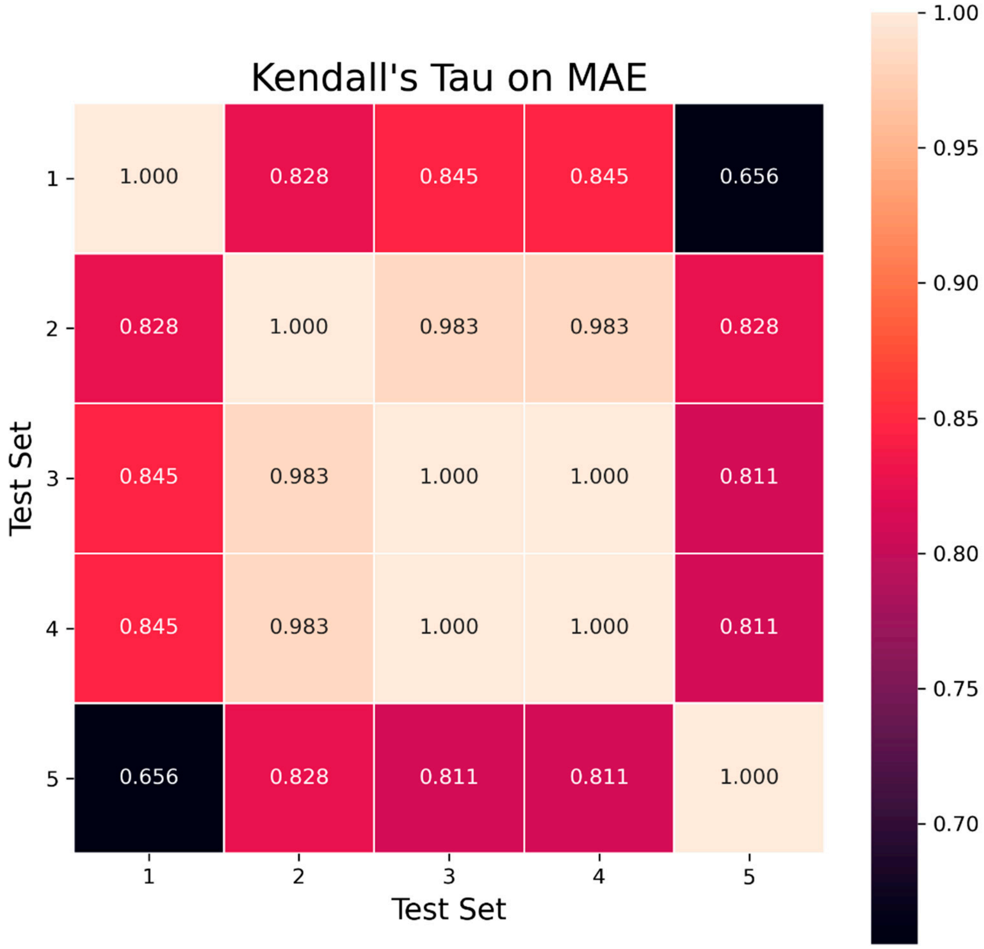

Figure S12: Heat map representing the Kendall's tau on the 5 test sets (numbered from 0 to 4) during repeated cross-validation. The correlation of the MAE results between the different test sets is very strong, always higher than 0.8, except between the test set 1 and 5 with a correlation of 0.6.

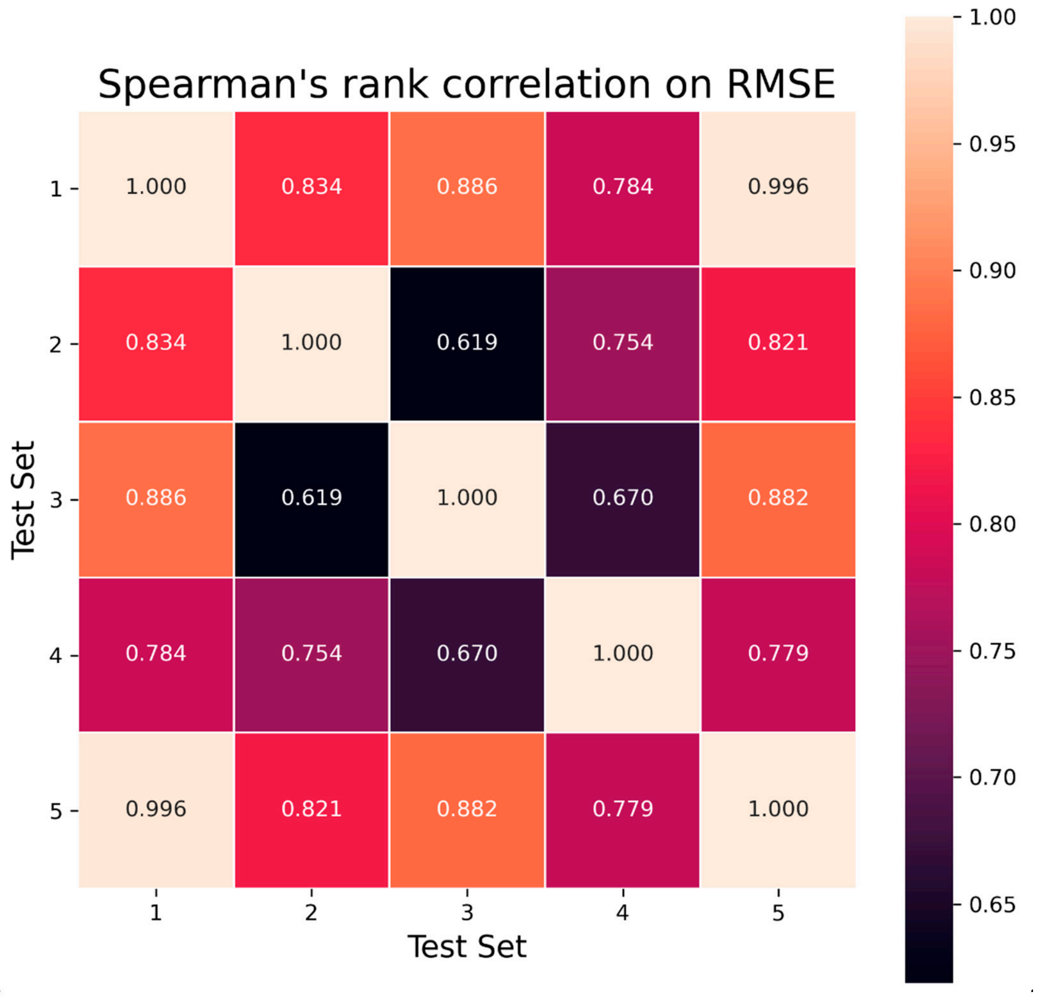

Figure S13: Heat map representing Spearman's rank correlation on the 5 test sets (numbered from 0 to 4) during repeated cross-validation. The correlation of the RMSE results between the different test sets is not very consistent with correlation values ranging from 0.6 to 0.9.

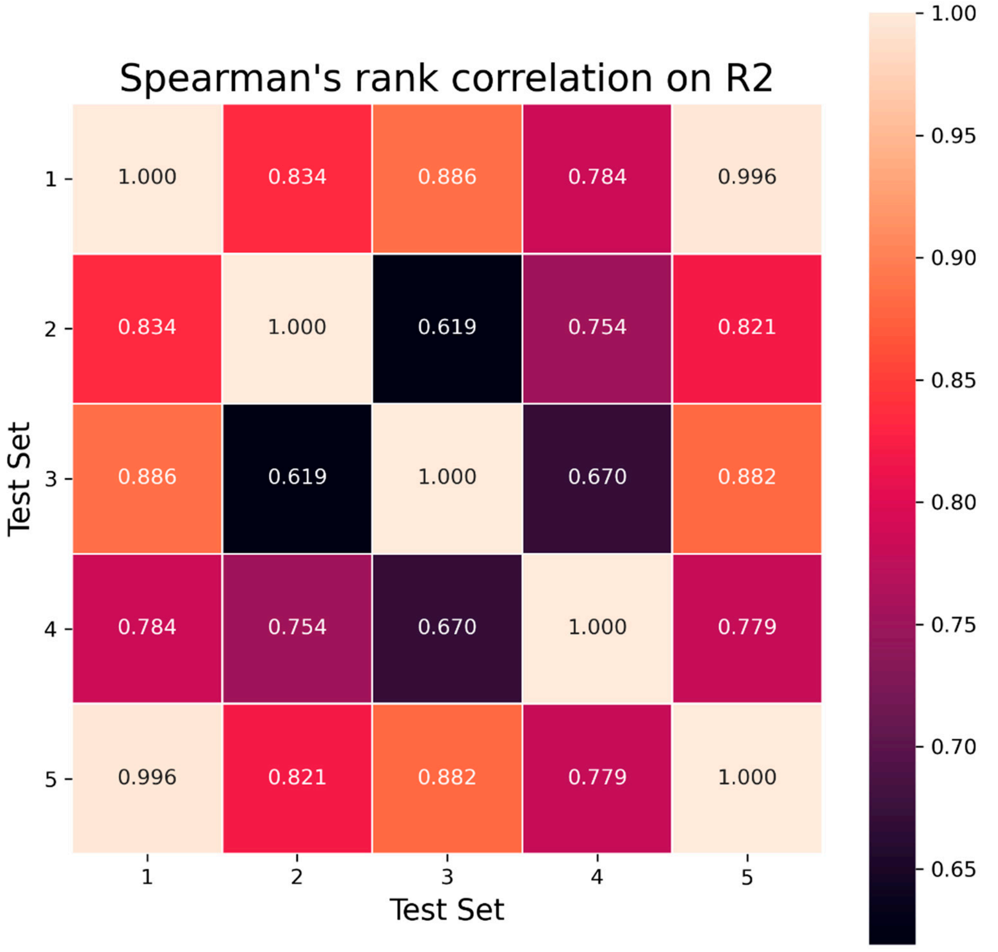

Figure S14: Heat map representing Spearman's rank correlation on the 5 test sets (numbered from 0 to 4) during repeated cross-validation. The correlation of the R^2 results between the different test sets is not very consistent with correlation values ranging from 0.6 to 0.9.

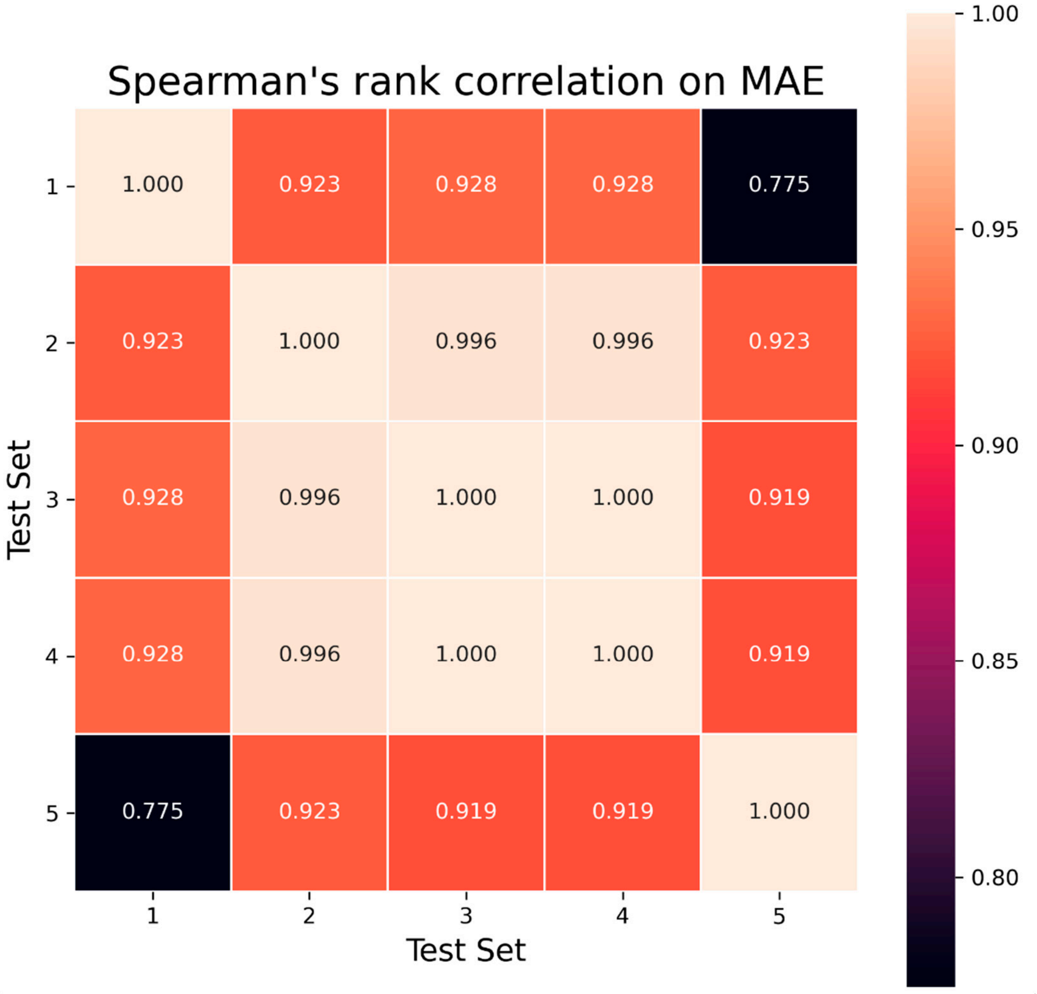

Figure S15: Heat map representing Spearman's rank correlation on the 5 test sets (numbered from 0 to 4) during repeated cross-validation. The correlation of the MAE results between the different test sets is very strong (always higher than 0.7).

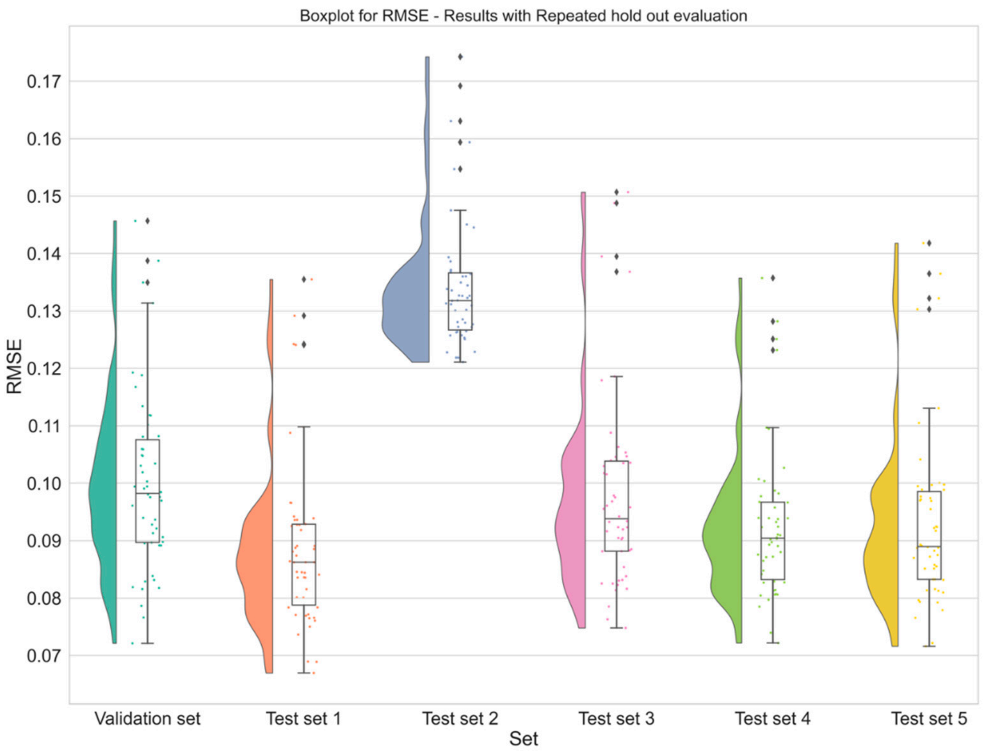

Figure S16: RMSE results of the 50 trained models with the repeated holdout evaluation procedure. Each block consists of a boxplot along with the density of results for each dataset (validation and test). The results vary essentially between 0.07 and 0.15. The results on the test sets are comparable and similar to the results on the validation set.

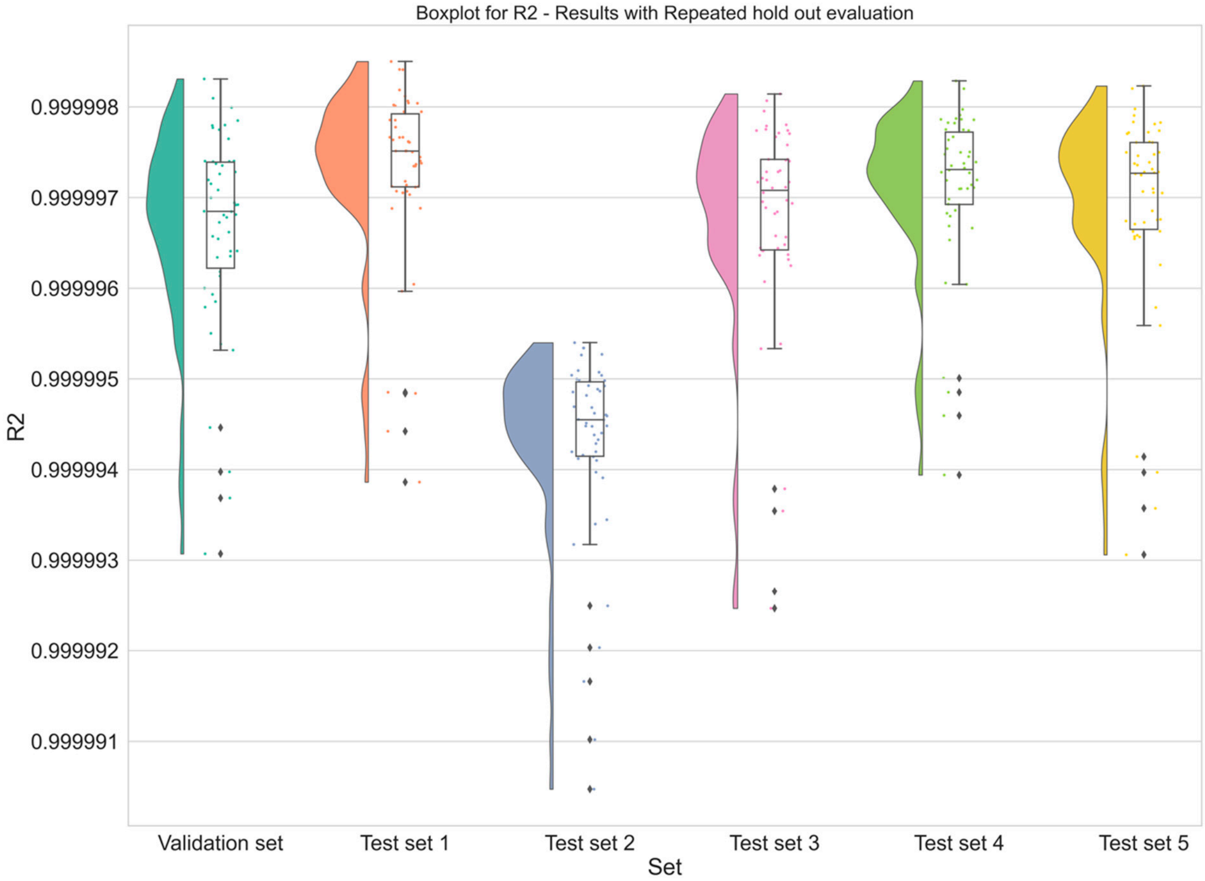

Figure S17: R^2 results using repeated holdout evaluation on the validation and the 5 test sets. For each plot, a boxplot with the density distribution is used to represent the R^2 value of each of the 50 trained models. The R^2 results obtained on the validation set and on the 5 test sets are higher than 0.999.

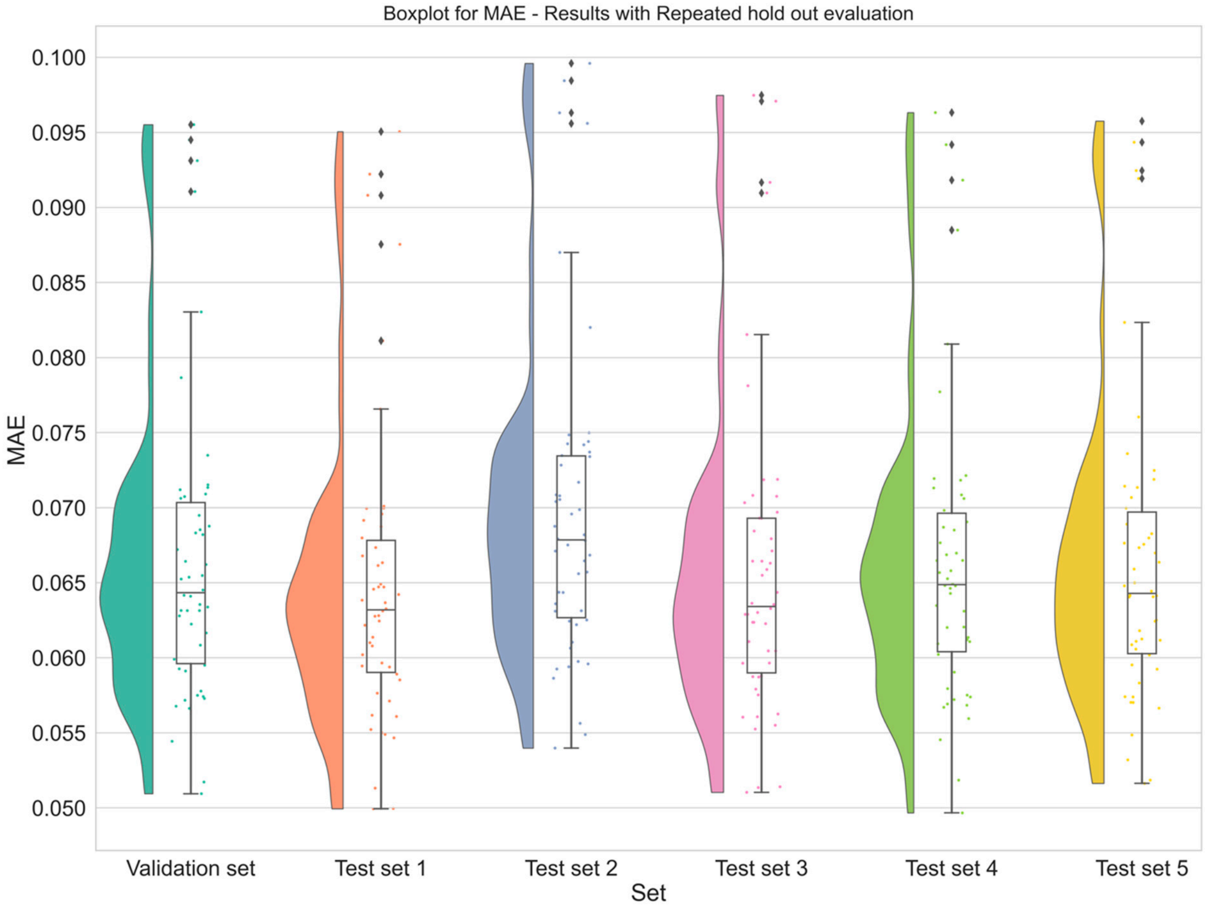

Figure S18: MAE results using repeated holdout evaluation on the validation and the 5 test sets. For each plot, a boxplot with the density distribution is used to represent the MAE value of each of the 50 trained models. The MAE results obtained on the validation set and on the 5 test sets range between 0.05 and 0.10.

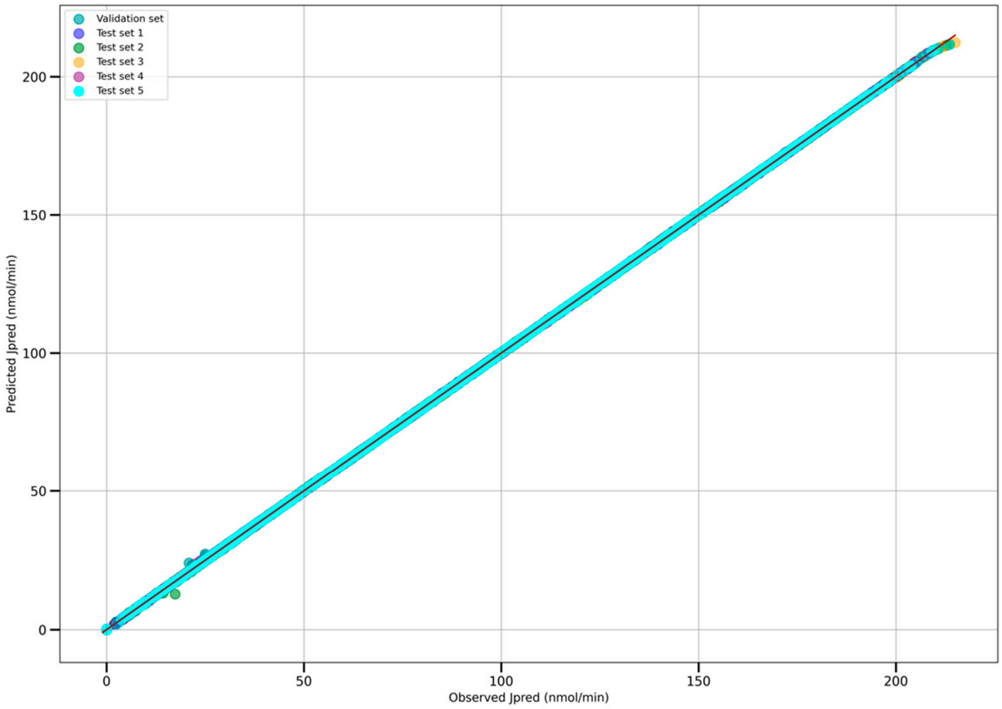

Figure S19: In this figure, a random model is picked in the repeated hold-out evaluation procedure to show the difference between the predicted and expected target for the validation set and the test sets. Except for a few instances, the predictions are almost similar to the expected value. This demonstrates, for the given model, good generalization capacity.

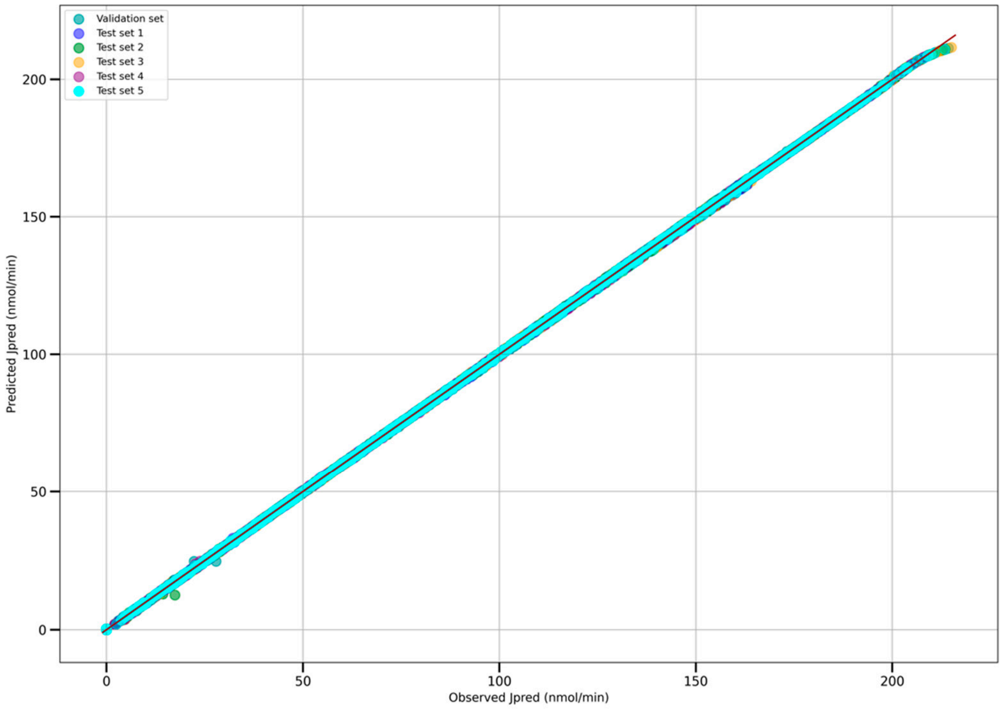

Figure S20: Similarly to Figure S19, this plot shows the difference between the predicted and observed output from the validation set and the test sets. This time, another random model from the repeated hold-out evaluation approach is selected to generate this figure. While a different model is used, the figure is almost identical to Figure S19, and this similarity is found when using any other model from this approach. This justifies the great generalization capabilities of models from the repeated hold-out evaluation approach.

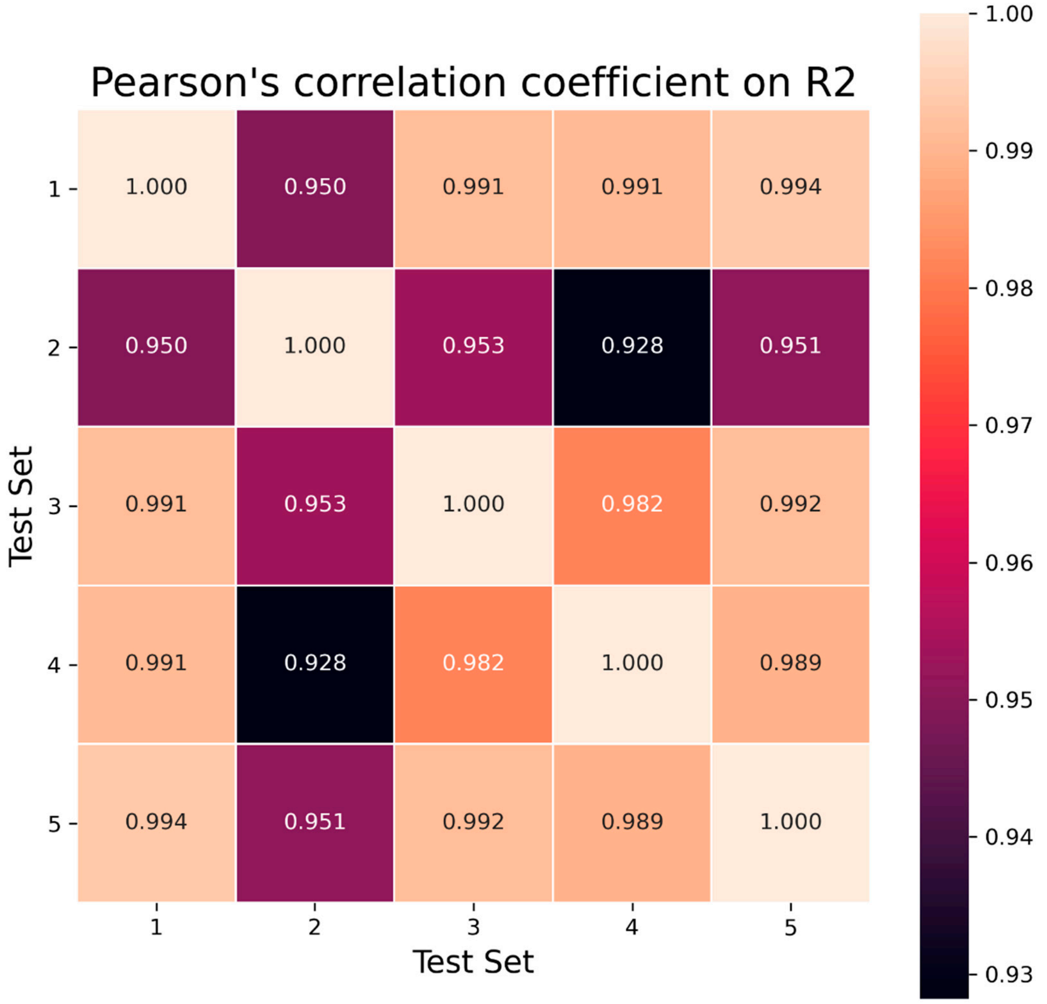

Figure S21: Heat map representing Pearson's correlation coefficient on the 5 test sets (numbered from 0 to 4) during repeated holdout evaluation. The correlation of the R^2 results between the different test sets is very strong (always higher than 0.9): The models have a good generalization capacity.

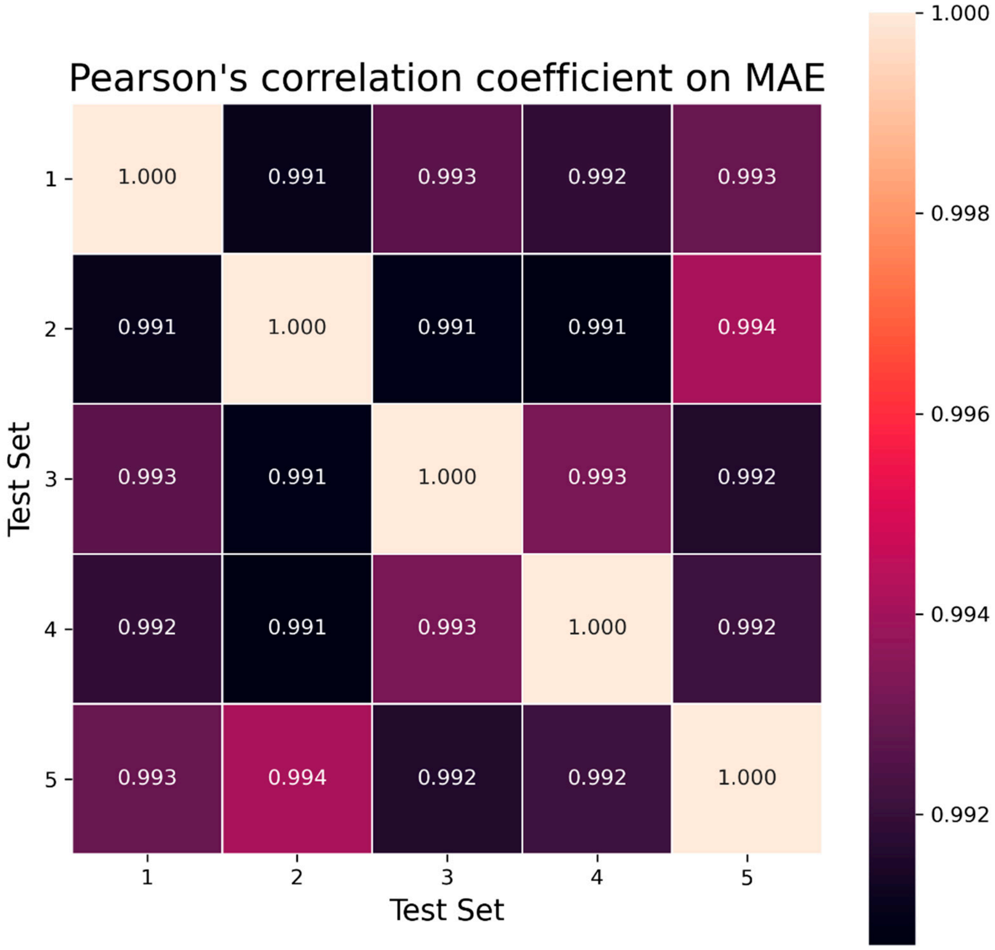

Figure S22: Heat map representing Pearson's correlation coefficient on the 5 test sets (numbered from 0 to 4) during repeated holdout evaluation. The correlation of the MAE results between the different test sets is very strong (always higher than 0.9): The models have a good generalization capacity.

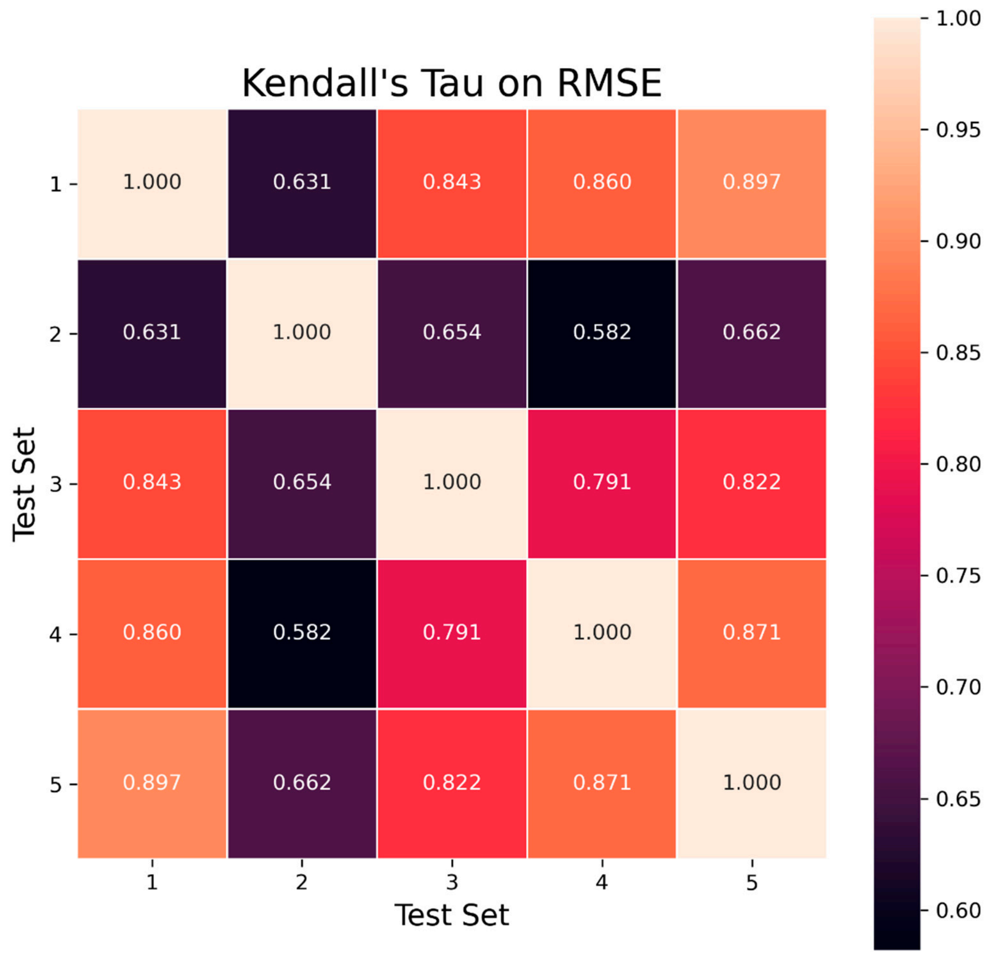

Figure S23: Heat map representing the Kendall's tau on the 5 test sets (numbered from 0 to 4) during repeated holdout evaluation. The correlation of the RMSE results between the different test sets is not very consistent, ranging from 0.5 to 0.8.

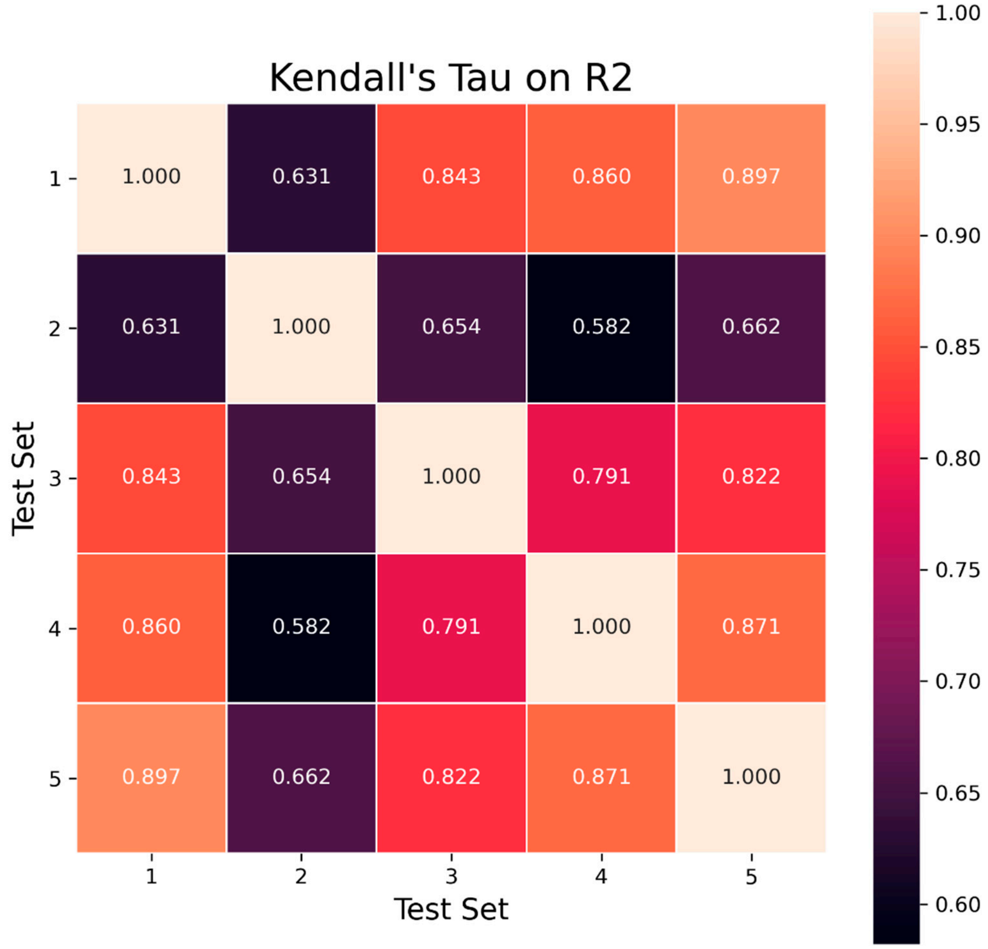

Figure S24: Heat map representing the Kendall's tau on the 5 test sets (numbered from 0 to 4) during repeated holdout evaluation. The correlation of the R^2 results between the different test sets is not very consistent, ranging from 0.5 to 0.8.

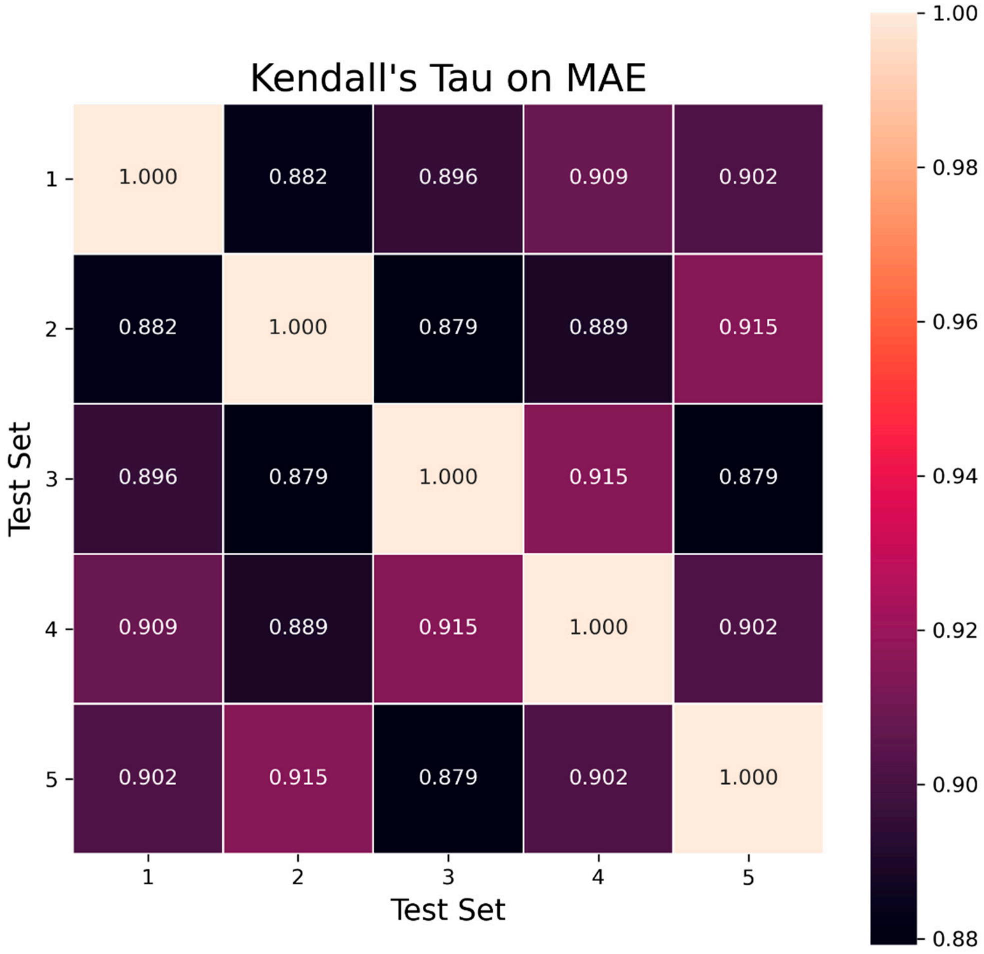

Figure S25: Heat map representing the Kendall's tau on the 5 test sets (numbered from 0 to 4) during repeated holdout evaluation. The correlation of the MAE results between the different test sets is very strong (always higher than 0.8).

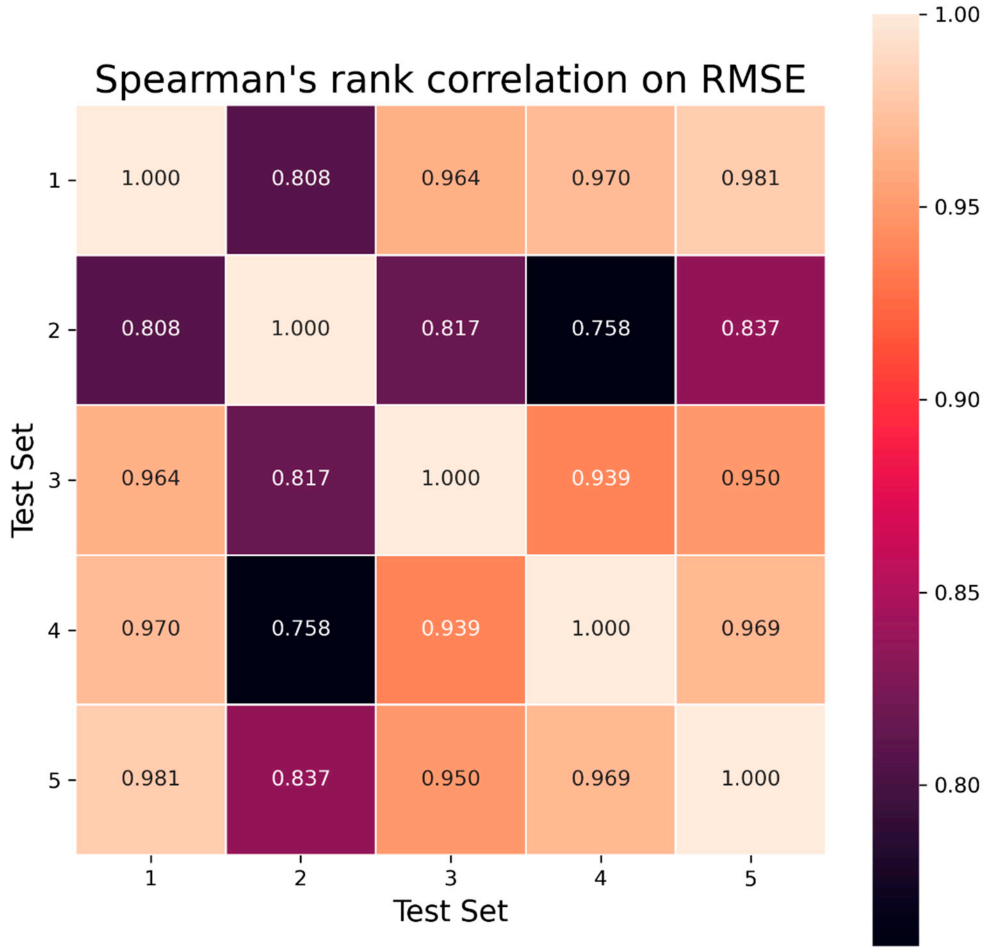

Figure S26: Heat map representing Spearman's rank correlation on the 5 test sets (numbered from 0 to 4) during repeated holdout evaluation. The correlation of the RMSE results between the different test sets is strong with correlation values higher than 0.7.

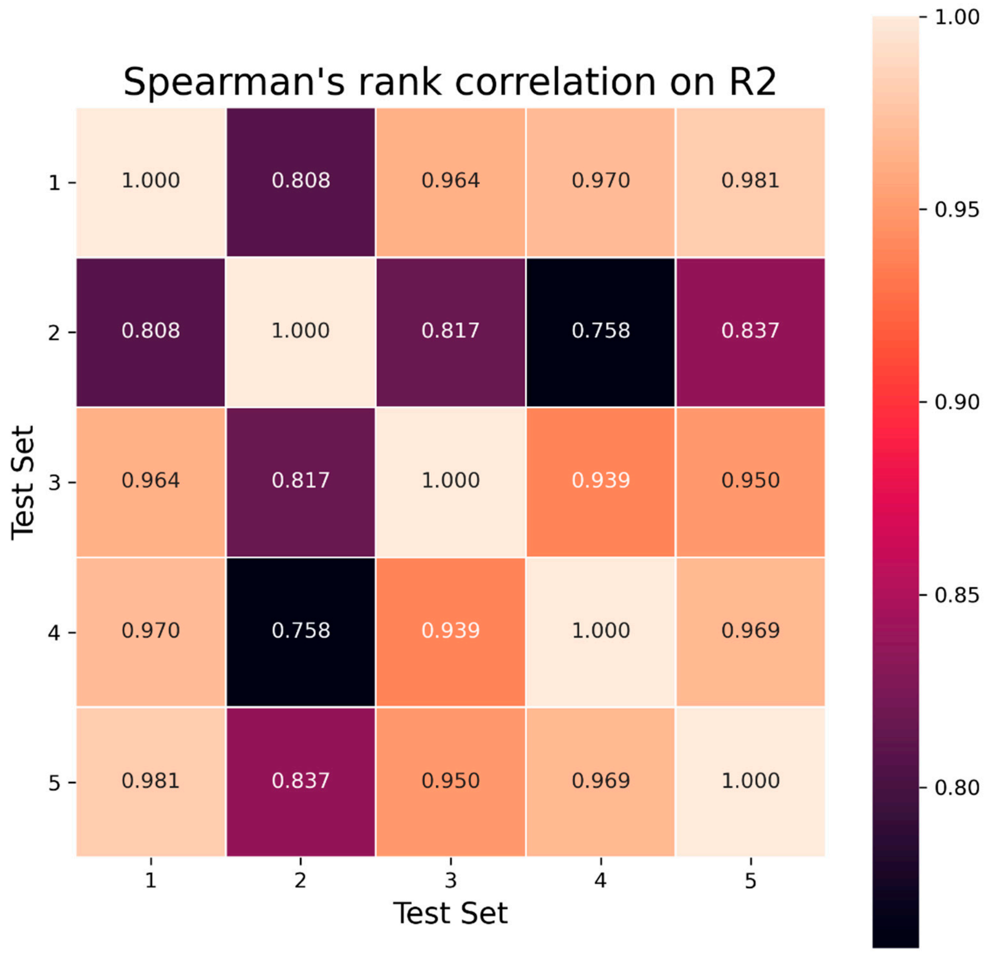

Figure S27: Heat map representing Spearman's rank correlation on the 5 test sets (numbered from 0 to 4) during repeated holdout evaluation. The correlation of the R^2 results between the different test sets is strong with correlation values higher than 0.7.

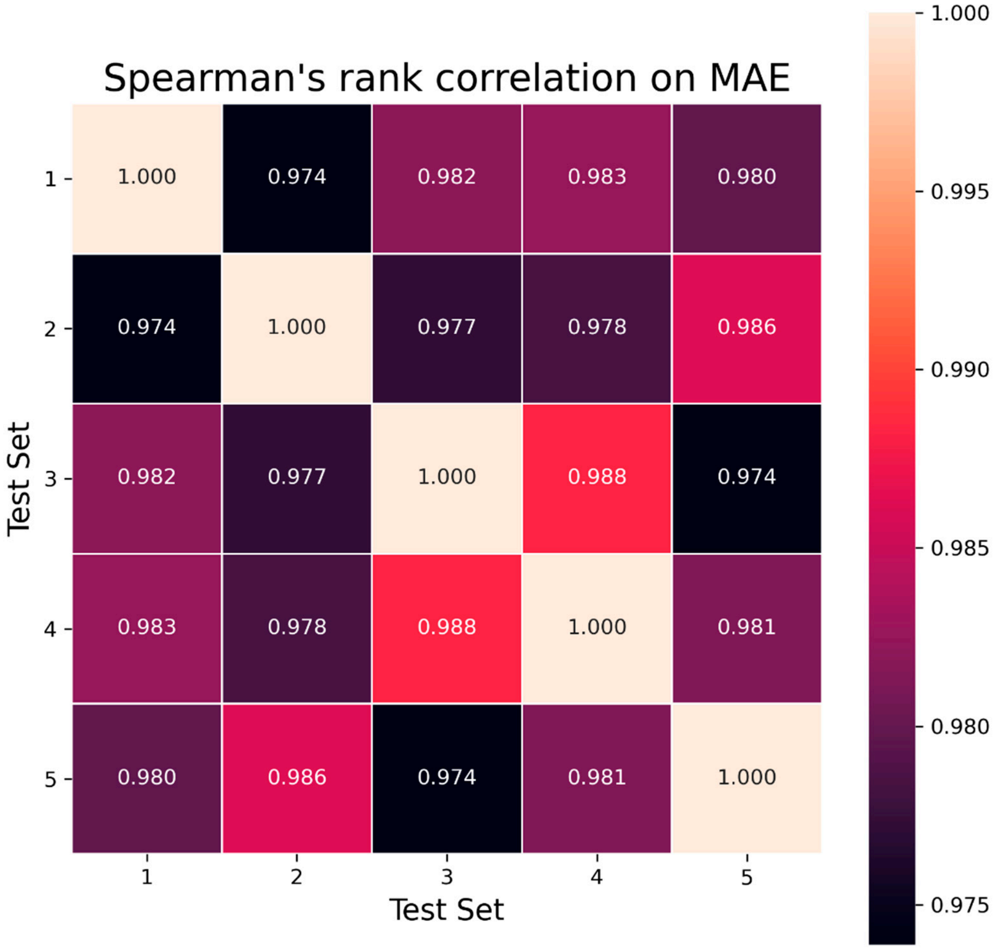

Figure S28: Heat map representing Spearman's rank correlation on the 5 test sets (numbered from 0 to 4) during repeated holdout evaluation. The correlation of the MAE results between the different test sets is very strong with correlation values always higher than 0.9.

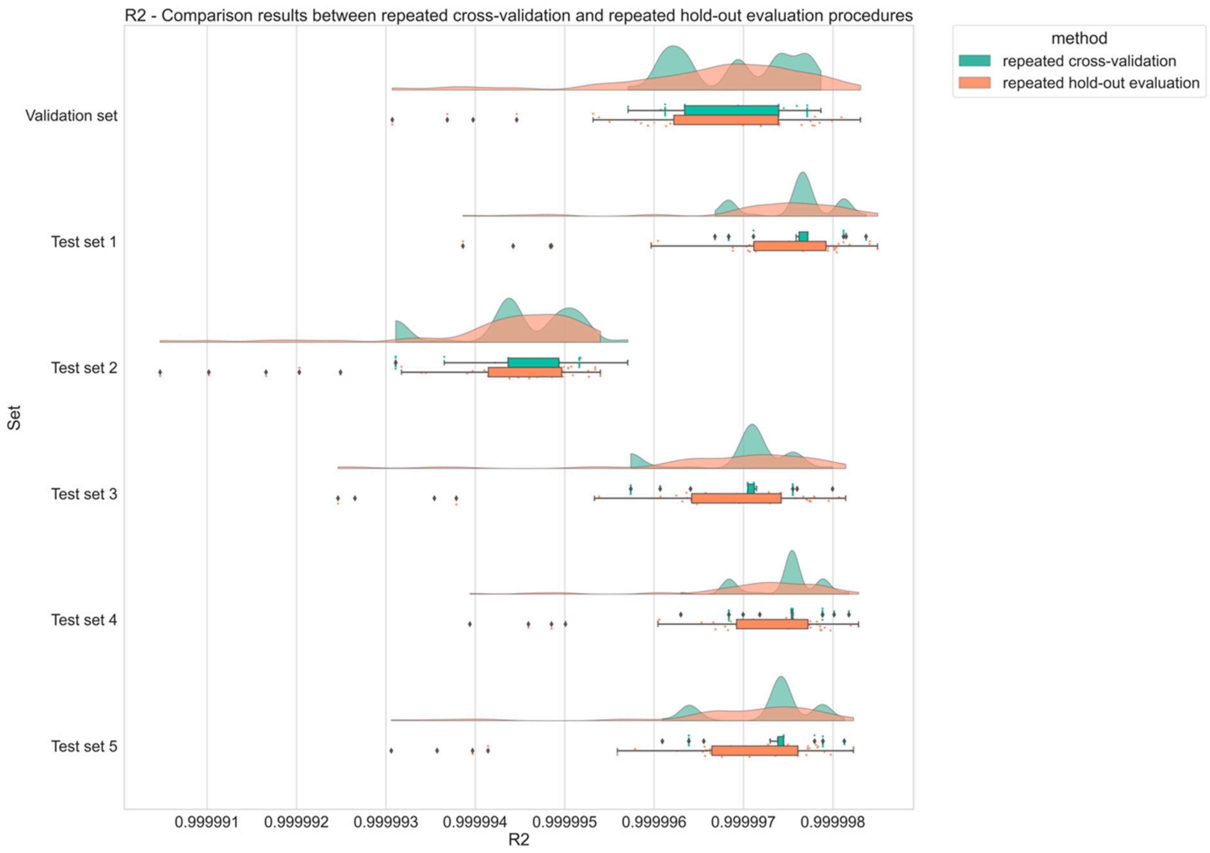

Figure S29: Overlay of the R^2 boxplots obtained during the two procedures (repeated cross validation and repeated holdout evaluation) to better observe the differences in performance, if any. Find in green the performance of cross-validation models and in orange

repeated hold-out evaluation models. Their results are very similar.

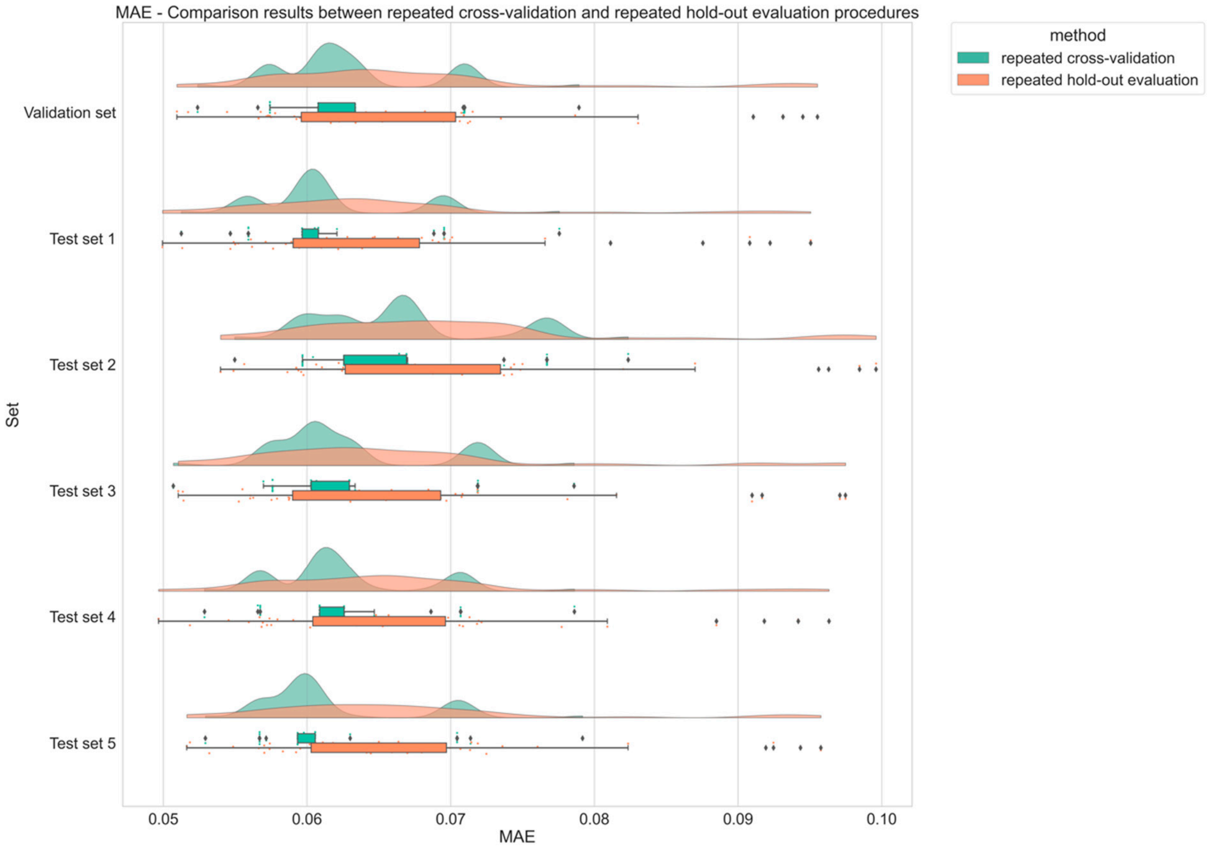

Figure S30: Overlay of the MAE boxplots obtained during the two procedures (repeated cross validation and repeated holdout evaluation) to better observe the differences in performance if any. Find in green the performance of cross-validation models and in orange repeated hold-out evaluation models. Their results are very similar.

Table S3. Instances with features and targets coming from the experimental wet lab dataset.

PGAM	ENO	PPDK	Jmeas
0	328.5	196.5	0
36.0151	328.5	196.5	17.3732
51.0526	328.5	196.5	19.1665
58.0451	328.5	196.5	22.8164
62.9323	328.5	196.5	21.4759
70.0001	328.5	196.5	22.5015
75.1128	328.5	196.5	25.1747
83.0827	328.5	196.5	24.9647
90.0752	328.5	196.5	28.9749
108.1955	328.5	196.5	31.9501
75	0	196.5	0
75	71.7831	196.5	14.2986
75	143.2672	196.5	21.0699
75	200.7381	196.5	20.9467
75	250.8957	196.5	21.6866
75	286.4863	196.5	20.8769
75	328.4225	196.5	22.2502
75	372.6276	196.5	24.2554
75	458.3621	196.5	24.9327
75	328.5	0	0
75	328.5	77.5352	18.3913
75	328.5	115.8959	21.8478
75	328.5	134.9498	22.6304
75	328.5	155.1837	22.1087
75	328.5	174.2521	24.7174
75	328.5	186.1501	23.8696
75	328.5	197.0864	21.8478
75	328.5	213.2924	23.8043
75	328.5	232.1341	27.84782

References

1. Bergstra, J.; Bardenet, R.; Bengio, Y.; Kégl, B. Algorithms for Hyper-Parameter Optimization. In Proceedings of the Advances in Neural Information Processing Systems; Curran Associates, Inc., 2011; Vol. 24.
2. Bergstra, J.; Bengio, Y. Random Search for Hyper-Parameter Optimization.
3. Kiefer, J.; Wolfowitz, J. Stochastic Estimation of the Maximum of a Regression Function. *The Annals of Mathematical Statistics* **1952**, *23*, 462–466.
4. Tieleman, T.; Hinton, G. Lecture 6.5-Rmsprop: Divide the Gradident by a Running Average of Its Recent Magnitude. *COURSERA: Neural networks for machine learning* 2012, 26–31.
5. Kingma, D.P.; Ba, J. Adam: A Method for Stochastic Optimization. *arXiv:1412.6980 [cs]* **2017**.
6. Rumelhart, D.E.; Hinton, G.E.; Williams, R.J. Learning Representations by Back-Propagating Errors. *Nature* **1986**, *323*, 533–536, doi:10.1038/323533a0.

7. Prechelt, L. Early Stopping - But When? In *Neural Networks: Tricks of the Trade*; Orr, G.B., Müller, K.-R., Eds.; Lecture Notes in Computer Science; Springer Berlin Heidelberg: Berlin, Heidelberg, 1998; Vol. 1524, pp. 55–69 ISBN 978-3-540-65311-0.
8. Srivastava, N.; Hinton, G.; Krizhevsky, A.; Sutskever, I.; Salakhutdinov, R. Dropout: A Simple Way to Prevent Neural Networks from Overfitting. *The journal of machine learning research* **2014**, *15*, 1929–1958.
9. Clevert, D.-A.; Unterthiner, T.; Hochreiter, S. Fast and Accurate Deep Network Learning by Exponential Linear Units (ELUs) 2016.
